# Edge-Aware Short-Chain Diffusion Enables High-Fidelity Sparse-Sampling Optoacoustic Tomography

**DOI:** 10.34133/bmef.0292

**Published:** 2026-07-23

**Authors:** Ying Fan, Ting Feng, Yangkun Liu, Abdula Aji, Libo Jiang, Dean Ta

**Affiliations:** ^1^ College of Future Information Technology, Fudan University, Shanghai 200433, China.; ^2^ Yiwu Research Institute of Fudan University, Zhejiang 322000, China.; ^3^ Department of Orthopaedic Surgery, Zhongshan Hospital, Fudan University, Shanghai 200032, China.; ^4^ Department of Rehabilitation Medicine, Huashan Hospital, Fudan University, Shanghai, China.

## Abstract

**Objective:** To improve image quality in multispectral optoacoustic tomography (MSOT) under conditions of strong noise and extreme sparse sampling. **Impact Statement:** This work provides a practical solution to enhance MSOT image quality while reducing hardware requirements, which may help expand its use in preclinical and clinical imaging. **Introduction:** MSOT provides useful biochemical and molecular contrast in tissues. However, image quality is often limited by system noise and sparse sampling. **Methods:** We propose the optoacoustic universal denoising network (OA-UDNet), a hybrid diffusion-based framework for sparse MSOT data. The model is trained on more than 250,000 in vivo images. It performs joint denoising and high-fidelity image restoration by combining an edge-aware module with diffusion-based generation to preserve structural boundaries. **Results:** With data from only 32 detectors, the method improves image quality and increases peak signal-to-noise ratio (PSNR) by nearly 14 dB. Evaluated against the standard 256-detector reference, the framework consistently preserves structural fidelity under extreme undersampling. Validation on whole-body mouse imaging, tumor models, and human samples shows reduced artifacts and improved recovery of anatomical and functional features. **Conclusion:** OA-UDNet improves MSOT image quality under sparse conditions. It offers a simple and effective way to accelerate imaging while reducing hardware complexity.

## Introduction

Optoacoustic imaging (OAI), which exploits laser-induced photoacoustic effects in biological tissues, overcomes the limitations of optical scattering, enabling high-resolution visualization of deep tissue structures [1,2]. Among OAI modalities, multispectral optoacoustic tomography (MSOT) integrates optical and ultrasound technologies, providing comprehensive anatomical, functional, and molecular information with enhanced tissue penetration and spatial resolution [1,3,4]. Its noninvasive, radiation-free nature further supports longitudinal monitoring, with demonstrated utility in tumor diagnosis [5], therapeutic evaluation [6], vascular imaging [7], and neuroscience [8]. Importantly, MSOT enables molecular functional imaging, facilitating real-time monitoring of critical parameters such as tumor biomarkers and oxygen metabolism, making it a promising tool for personalized medicine and early cancer detection.

Despite these advantages, the clinical translation of MSOT remains hampered by several technical challenges [9]. Conventional systems rely on dense sensor arrays and full-view acquisition, which increase hardware complexity and cost while limiting image reconstruction speed [9]. Sparse-view imaging has emerged to mitigate these constraints [1], but the resulting data insufficiency introduces severe noise, aliasing artifacts, and reduced resolution, heavily compromising diagnostic quality [10]. Consequently, achieving high-quality image reconstruction under extreme sparsity remains a major challenge. Although deep learning, especially U-Net-based models, has improved MSOT reconstruction by reducing artifacts [11], most existing methods treat denoising and image restoration as separate tasks [12–15]. Recently, the diffusion models have set new benchmarks in medical image restoration by coupling learned forward-backward processes with iterative reconstruction [15]. However, standard latent diffusion models (LDMs) often struggle with the extreme structural degradation of highly sparse MSOT, and their prolonged iterative sampling limits practical deployment. Furthermore, the task separation in existing studies often overlooks the intrinsic interplay between noise suppression and high-frequency detail recovery, limiting the overall image quality under extreme sparsity.

To address these challenges, we propose the optoacoustic universal denoising network (OA-UDNet), a hybrid diffusion framework for joint denoising and high-fidelity image restoration. To our knowledge, this is the first method to perform both tasks together under extreme OAI sparsity within a unified generative model. Unlike standard LDMs [16,17], OA-UDNet uses a hybrid dual-space refinement design. The entire forward propagation logic is explicitly organized into 3 sequential stages. Stage 1: Feature compression and initial reconstruction. The sparse input image tensor is encoded into a bottleneck latent feature and subsequently decoded to produce a dense initial reconstructed image. Stage 2: Iterative image-space diffusion loop. A short-chain diffusion process with only 10 steps is applied directly to this initial reconstructed image tensor in the spatial domain. Stage 3: Final generation. The denoised image is passed through the shared encoder–decoder one final time to enforce structural consistency with the target MSOT data.

To preserve important anatomical boundaries during the short diffusion process, we include an edge-aware self-attention (EAS) module in the denoising U-Net. Unlike standard attention methods that can fit to noise, EAS uses a fixed Sobel prior together with an affine grid sampling network. This helps maintain geometric alignment and clear edges without introducing unstable gradients under strong noise. By combining this structural prior with fast diffusion and dual-space regularization, OA-UDNet produces stable and high-quality reconstructions even under severely ill-posed conditions, and outperforms conventional single-task models.

## Results

### Development and characterization of OA-UDNet

**Fig. 1.**
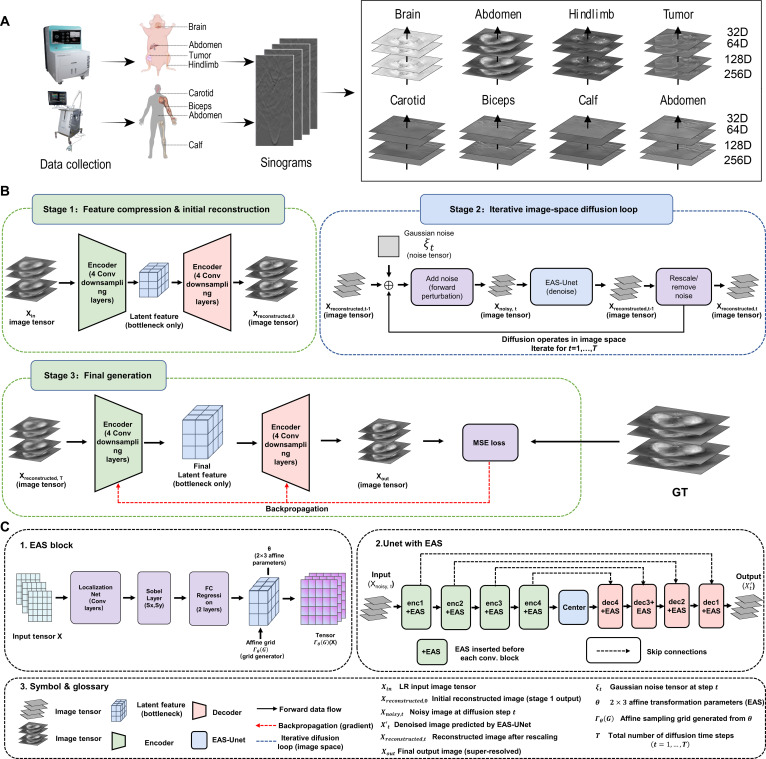
Overview of the proposed OA-UDNet framework. (A) Paired sparse-sampling inputs (32-, 64-, and 128-detector) and 256-detector full-view ground truth preparation across diverse murine organs and human tissues. (B) End-to-end training pipeline structured into 3 sequential stages: stage I for initial reconstruction via the encoder–decoder, stage II for the iterative image-space diffusion loop, and stage III for final high-fidelity generation. (C) Core architectural components illustrating the EAS block and EUNet configuration, complemented by a comprehensive symbol glossary defining all image tensors, latent representations, and operational data flows.

The overall workflow of the proposed OA-UDNet is shown in Fig. 1. The framework uses a hybrid dual-space refinement design to address the strong ill-posed nature of sparse-view MSOT reconstruction. During inference, the degraded sparse input is first mapped to a dense initial estimate through an encoder–decoder. Instead of using a long latent diffusion process, this estimate is refined in the image space with a fast 10-step diffusion process. At each step, the edge-aware U-Net (EUNet) performs noise reduction and recovers high-frequency details. The embedded EAS module uses fixed Sobel priors to preserve structural boundaries and prevent false features under strong noise. The result is then passed through the encoder–decoder again to enforce consistency with the optoacoustic signal. The full model runs in a deterministic way and does not require extra tuning for new data, showing stable performance across different acquisition settings.

### Validation of structural fidelity via physical phantoms

Prior to in vivo application, we evaluated the model’s structural fidelity and the validity of our global speed-of-sound assumption using a customized physical phantom under the 32-detector configuration. Because the exact geometric properties of the physical phantom are well-defined, it serves as a reliable reference for identifying potential generative artifacts. The results demonstrated steady geometric recovery and aliasing artifact suppression, avoiding the generation of spurious pseudo-branches. Under this sparse condition, OA-UDNet achieved a PSNR of 37.35 dB and a structural similarity index (SSIM) of 0.986 (Fig. S1 and Table S1), indicating that the framework effectively preserves physical boundaries under simplified physical conditions. However, we note that while these phantom results support geometric fidelity, they do not definitively exclude the possibility of hallucinated structures in more complex biological scenarios.

### Evaluation of OA-UDNet for mouse datasets

**Fig. 2.**
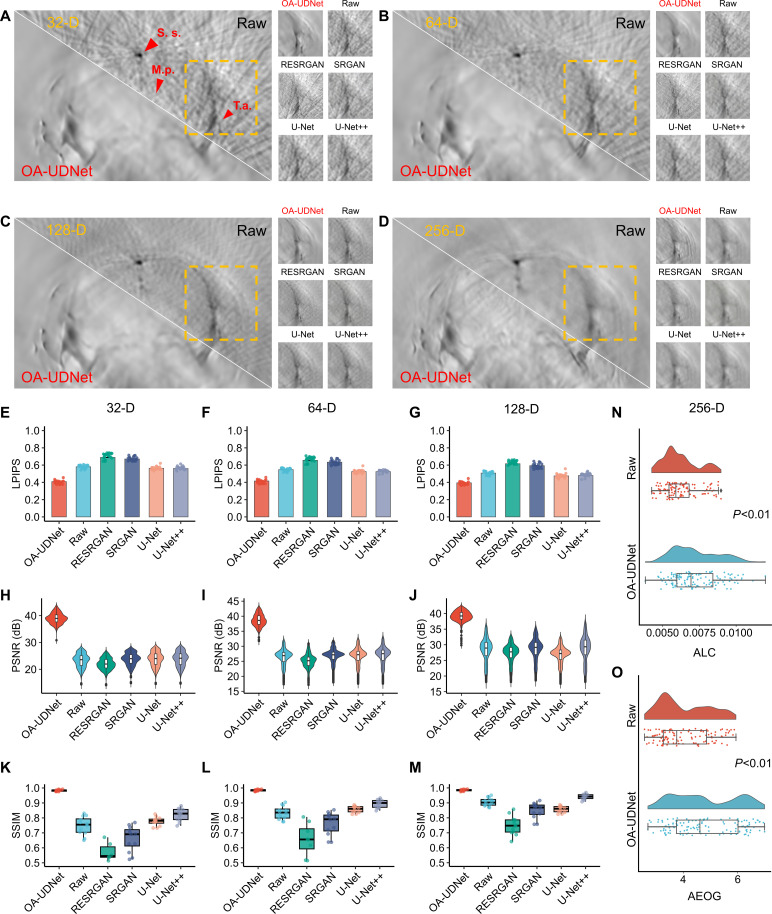
Comparative analysis of OA-UDNet with benchmark methods on the mouse brain dataset. (A to D) Visual comparison of OAI enhancement in the brain region achieved by OA-UDNet and 4 methods: Real-ESRGAN, SRGAN, U-Net, and U-Net++ at 800 nm. Yellow boxes emphasize local structural details of the brain. S.s., sagittal sinus; M.p., mesencephalon; T.a., temporal artery. (E to G) Quantitative assessment of LPIPS metrics, highlighting the superior performance of OA-UDNet compared to the 4 methods on brain OAI data. (H to J) Violin–scatter plots showing PSNR distributions for OA-UDNet and competing methods across 32- to 128-detector arrays in the brain region. (K to M) Box plots illustrating SSIM improvements achieved by OA-UDNet over the 4 methods across 32- to 128-detector arrays for brain OAI data. (N and O) Raincloud plots comparing ALC and AEOG metrics between raw and OA-UDNet-enhanced OAI for 256-detector arrays in the brain region, demonstrating significant improvements in image detail and contrast.

**Fig. 3.**
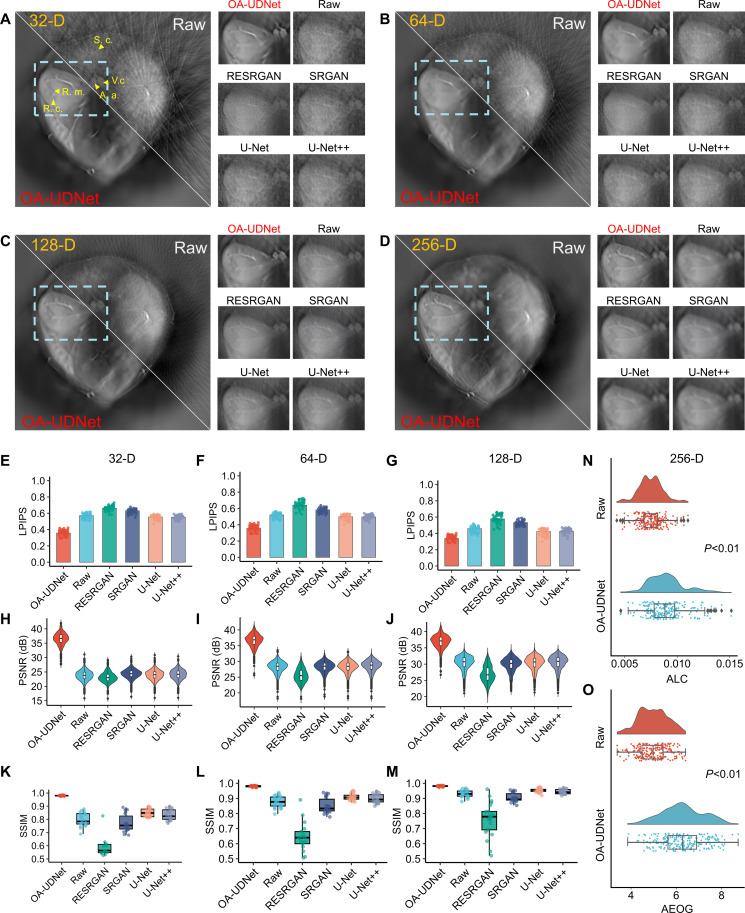
Comparative analysis of OA-UDNet with benchmark methods on the mouse abdominal dataset. (A to D) Visual comparison of optoacoustic image (OAI) enhancement in the abdominal region at 800 nm, produced by OA-UDNet and 4 benchmark methods: Real-ESRGAN, SRGAN, U-Net and U-Net++. Blue boxes highlight local anatomical details: R.m. (renal medulla), R.c. (renal cortex), S.c. (spinal cord), V.c. (vena cava), and A.a. (abdominal aorta). (E to G) Quantitative evaluation of LPIPS metrics for the abdominal dataset, highlighting the superior performance of OA-UDNet compared to the 4 benchmark methods. (H to J) Violin–scatter plots displaying PSNR distributions for OA-UDNet and the competitive methods across 32- to 128-detector arrays in the abdominal region. (K to M) Box plots illustrating SSIM improvements achieved by OA-UDNet over the 4 methods across 32- to 128-detector arrays for the abdominal dataset. (N and O) Raincloud plots comparing ALC and AEOG metrics between raw OAI and OA-UDNet-enhanced images for 256-detector arrays in the abdominal region, showing substantial improvements in image detail and contrast.

**Fig. 4.**
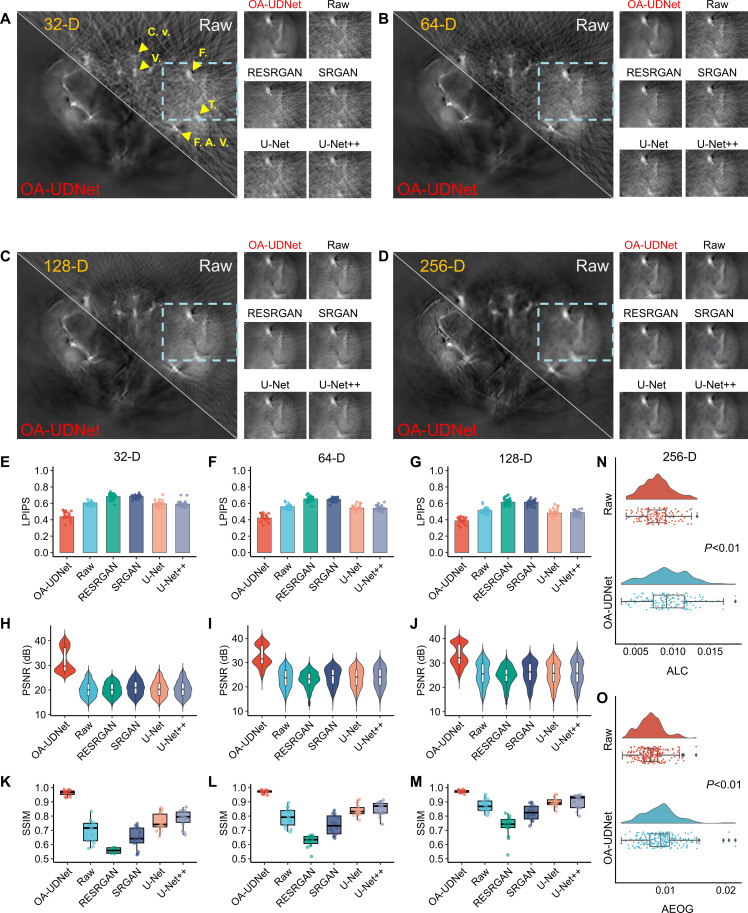
Comparative analysis of OA-UDNet and benchmark methods on the mouse hindlimb dataset. (A to D) Representative optoacoustic image enhancements in the mouse hindlimb dataset obtained using OA-UDNet and 4 benchmark methods (Real-ESRGAN, SRGAN, U-Net, and U-Net++) at 800 nm. C.v., caudal vertebrae; V., vein; F., fibula; T., tibia; F.A.V., femoral artery and vein. (E to G) Quantitative assessment of LPIPS values, highlighting the superior perceptual performance of OA-UDNet compared with the 4 benchmark methods. (H to J) Violin–scatter plots showing PSNR distributions for OA-UDNet and the benchmark methods across detector arrays ranging from 32 to 128 elements, demonstrating consistent improvements in reconstruction quality. (K to M) Box plots illustrating SSIM enhancements achieved by OA-UDNet relative to the benchmark methods under 32- to 128-detector configurations. (N and O) Raincloud plots comparing the ALC and AEOG between raw optoacoustic images and OA-UDNet-enhanced results using 256-detector arrays, emphasizing substantial gains in image detail and contrast.

**Fig. 5.**
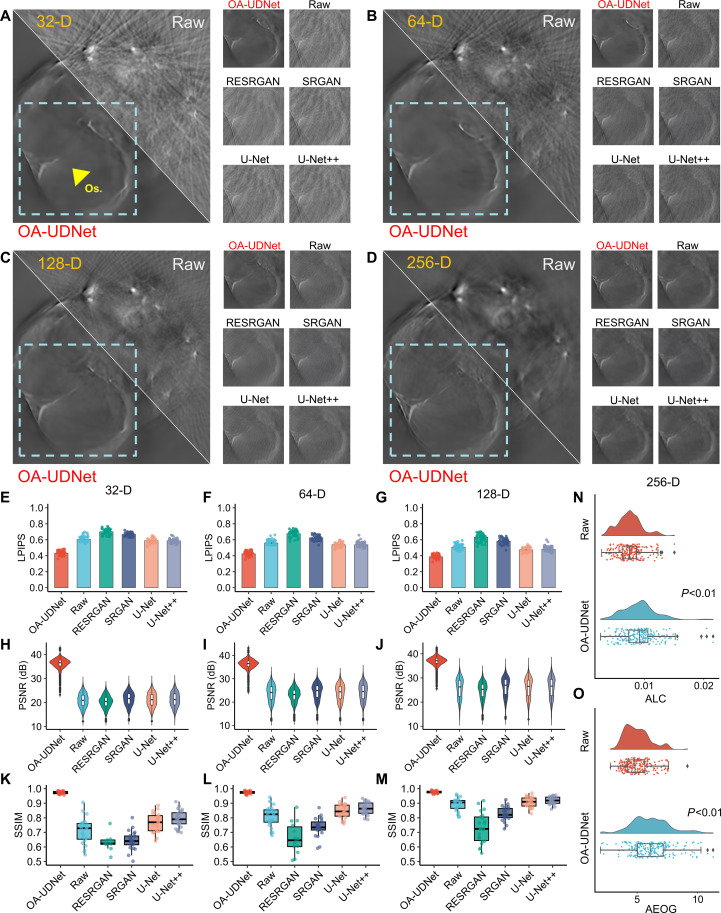
Comparative analysis of OA-UDNet with benchmark methods on the mouse osteosarcoma dataset. (A to D) Visual comparison of optoacoustic image enhancement in the mouse osteosarcoma dataset, obtained using OA-UDNet and 4 benchmark methods (Real-ESRGAN, SRGAN, U-Net, and U-Net++) at 800 nm. Os., osteosarcoma. (E to G) Quantitative evaluation of LPIPS metrics, highlighting the superior performance of OA-UDNet compared with the 4 benchmark methods on the osteosarcoma dataset. (H to J) Violin–scatter plots illustrating PSNR distributions for OA-UDNet and the benchmark methods across 32- to 128-detector arrays, demonstrating improved image quality in the osteosarcoma dataset. (K to M) Box plots showing SSIM improvements achieved by OA-UDNet relative to the 4 methods across 32- to 128-detector arrays for the osteosarcoma dataset. (N and O) Raincloud plots comparing ALC and AEOG metrics between raw OAI and OA-UDNet-enhanced images for 256-detector arrays, emphasizing significant enhancements in image detail and contrast within the osteosarcoma dataset.

We evaluated OA-UDNet on 4 representative in vivo mouse datasets (brain, abdomen, hindlimb, and tumor regions) acquired across a diverse array of configurations (32, 64, 128, and 256 detectors). The datasets comprised extensive samples per array: 1,212 for the brain, 2,172 for the abdomen, and a combined 3,616 for the hindlimb (1,353 healthy and 2,263 osteosarcoma samples). Under the sparsest 32-detector acquisitions, severe reconstruction artefacts obscured critical structures, rendering them largely indiscernible. Following enhancement with OA-UDNet, these artefacts were markedly attenuated, and internal structures and boundaries were restored. To comprehensively demonstrate the organ-agnostic robustness of our framework across varying acoustic attenuation and tissue scattering properties, we systematically present evaluations on distinctly different anatomical regions: the brain (Fig. 2), the abdomen (Fig. 3), the healthy hindlimb (Fig. 4), and the osteosarcoma tumor model (Fig. 5). In the sparsely sampled 32- and 64-detector reconstructions, where severe noise previously obscured fine structures, OA-UDNet effectively restored clear anatomical details across optically diverse tissues—such as the midbrain and temporal arteries in the brain, the spinal cord, renal cortex, medulla, and major vessels in the abdomen, and the hindlimb, fibula, and femoral vasculature in the hindlimb and tumor regions (Figs. 2A to D, 3A to D, 4A to D, and 5A to D). Fourier spectral analysis further confirmed the recovery of high-frequency spatial information (Figs. S2 to S5).

Quantitative assessments closely corroborate these qualitative improvements, as summarized in Table 1. Rather than exhibiting minor incremental changes, OA-UDNet yielded massive, statistically marked gains under the most extreme sparse-view scenarios. For instance, in the 32-detector acquisitions across the brain, abdomen, and hindlimb (both healthy and tumor-bearing) datasets, OA-UDNet consistently elevated PSNR from baseline levels of ~20 to 23 dB to ~31 to 39 dB (reflecting relative improvements of 62% to 77%). Concurrently, structural fidelity (SSIM) was restored from ~0.72 to 0.81 to near-ideal values of 0.96 to 0.98, while learned perceptual image patch similarity (LPIPS) perceptual errors were substantially reduced by ~27% to 33%. Comparable, robust quantitative improvements were consistently maintained at the 64-, 128-, and 256-detector densities (Notes S1 to S3).

**Table 1.** Performance comparison of different denoising and image restoration methods on in vivo datasets

Model	Datasets	32-D			64-D			128-D		
		PSNR↑	SSIM↑	LPIPS↓	PSNR↑	SSIM↑	LPIPS↓	PSNR↑	SSIM↑	LPIPS↓
OA-UDNet	Brain	38.987	0.983	0.408	38.676	0.983	0.416	38.737	0.984	0.395
	Abdomen	36.562	0.979	0.357	36.643	0.980	0.355	36.944	0.981	0.338
	Hindlimb	31.951	0.965	0.437	32.602	0.971	0.420	33.062	0.975	0.387
	Tumor	36.308	0.976	0.430	36.301	0.977	0.423	36.963	0.979	0.386
U-Net	Brain	23.755	0.806	0.563	26.641	0.876	0.525	28.530	0.876	0.475
	Abdomen	23.976	0.845	0.555	28.183	0.908	0.498	30.230	0.950	0.422
	Hindlimb	20.416	0.770	0.593	23.707	0.850	0.542	25.645	0.910	0.481
	Tumor	21.088	0.776	0.587	23.971	0.853	0.537	25.802	0.913	0.474
U-Net++	Brain	23.804	0.807	0.561	26.839	0.879	0.524	28.813	0.930	0.479
	Abdomen	24.101	0.850	0.552	28.481	0.913	0.495	30.384	0.951	0.427
	Hindlimb	20.482	0.775	0.591	23.900	0.857	0.541	25.850	0.914	0.486
	Tumor	21.220	0.781	0.586	24.176	0.859	0.537	25.991	0.916	0.479
SRGAN	Brain	23.804	0.642	0.671	26.672	0.745	0.634	28.588	0.834	0.593
	Abdomen	24.361	0.744	0.630	28.262	0.831	0.583	29.864	0.891	0.533
	Hindlimb	20.878	0.638	0.686	24.119	0.742	0.649	25.925	0.828	0.615
	Tumor	21.495	0.642	0.667	24.419	0.743	0.625	26.209	0.830	0.579
RESRGAN	Brain	22.024	0.754	0.689	25.044	0.599	0.656	27.225	0.754	0.616
	Abdomen	23.114	0.535	0.661	25.613	0.610	0.640	26.926	0.751	0.578
	Hindlimb	20.181	0.568	0.684	23.051	0.568	0.654	24.708	0.714	0.615
	Tumor	20.359	0.447	0.700	22.964	0.551	0.675	24.696	0.700	0.631

**Fig. 6.**
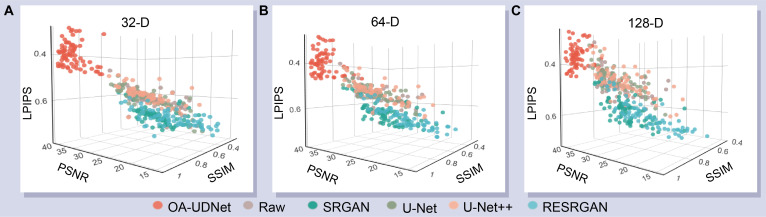
3D scatter plots and distribution plots comparing the quantitative performance of OA-UDNet, Real-ESRGAN, SRGAN, U-Net, and U-Net++ on mouse data acquired with 32, 64, and 128 detectors. (A) Quantitative evaluation utilizing a sparse 32-detector configuration. (B) Evaluation metrics obtained using a 64-detector array. (C) Performance under a high-density 128-detector.

As shown in Fig. 6, OA-UDNet consistently outperformed contemporary single-task image denoising and image restoration baselines (Real-ESRGAN, SRGAN, U-Net, and U-Net++) under sparse-view conditions (32 to 128 detectors). Across all arrays, it achieved higher PSNR and SSIM values alongside lower LPIPS scores (Figs. 2E to M, 3E to M, 4E to M, and 5E to M, *P* < 0.001; Tables S2 to S5). Furthermore, to evaluate the network’s behavior on full-view acquisitions, we applied OA-UDNet to the 256-detector data utilizing the aforementioned unsupervised identity mapping strategy. As visually demonstrated and quantitatively supported by higher average local contrast (ALC) and average energy of gradient (AEOG) scores (Fig. S6), this approach explicitly suppresses inherent background noise compared to the raw 256-detector baselines, reflecting the network’s inherent noise-suppression characteristics. These perceptual and metric improvements are comprehensively summarized in Figs. 2 to 5 (Figs. 2N and O, 3N and O, 4N and O, and 5N and O, *P* < 0.001). Together, these results demonstrate that OA-UDNet reliably recovers high-frequency information and structural detail across varying organs and sampling densities, supporting its generalizability for sparse-sampling OAI.

Finally, to assess multispectral performance, we evaluated the model’s generalization ability using data from 12 distinct wavelengths under sparse conditions (specifically across the 32-, 64-, and 128-detector configurations). Evaluations across these various detector arrays revealed that OA-UDNet significantly improved image quality in both the low and high optical wavelength regimes (see Figs. S7 to S10). Further quantitative analysis of the multispectral data in the test set demonstrated substantial improvement across all evaluation metrics following OA-UDNet processing when compared to the original OAI (see Table 2).

**Table 2.** Quantitative evaluation of LPIPS, PSNR, and SSIM metrics of OA-UDNet in mouse dataset

Wavelengths	Metrics	OA-UDNet (32-D)	Raw (32-D)	OA-UDNet (64-D)	Raw (64-D)	OA-UDNet (128-D)	Raw (128-D)
680 nm	PSNR	35.80 ± 2.70	21.23 ± 2.55	35.80 ± 2.49	24.48 ± 2.88	36.31 ± 2.49	26.55 ± 3.66
	SSIM	0.97 ± 0.01	0.70 ± 0.06	0.97 ± 0.01	0.79 ± 0.04	0.98 ± 0.01	0.88 ± 0.03
	LPIPS	0.42 ± 0.04	0.61 ± 0.03	0.41 ± 0.04	0.57 ± 0.02	0.39 ± 0.03	0.52 ± 0.03
700 nm	PSNR	35.48 ± 2.67	21.57 ± 2.43	36.02 ± 2.46	24.85 ± 2.82	36.48 ± 2.37	26.94 ± 3.59
	SSIM	0.97 ± 0.01	0.71 ± 0.06	0.97 ± 0.01	0.81 ± 0.04	0.98 ± 0.01	0.88 ± 0.03
	LPIPS	0.41 ± 0.04	0.60 ± 0.03	0.42 ± 0.03	0.56 ± 0.02	0.39 ± 0.03	0.51 ± 0.03
730 nm	PSNR	35.55 ± 2.64	21.57 ± 2.55	36.21 ± 2.48	24.89 ± 2.92	36.63 ± 2.38	26.94 ± 3.67
	SSIM	0.97 ± 0.01	0.72 ± 0.06	0.97 ± 0.01	0.81 ± 0.04	0.98 ± 0.01	0.89 ± 0.03
	LPIPS	0.40 ± 0.03	0.59 ± 0.03	0.41 ± 0.03	0.56 ± 0.02	0.39 ± 0.03	0.50 ± 0.02
760 nm	PSNR	35.65 ± 2.76	21.62 ± 2.55	36.26 ± 2.53	24.66 ± 2.96	36.62 ± 2.54	26.68 ± 3.79
	SSIM	0.97 ± 0.01	0.71 ± 0.06	0.97 ± 0.01	0.81 ± 0.05	0.98 ± 0.01	0.89 ± 0.03
	LPIPS	0.41 ± 0.03	0.59 ± 0.03	0.42 ± 0.03	0.55 ± 0.02	0.39 ± 0.03	0.51 ± 0.03
800 nm	PSNR	35.77 ± 2.68	22.06 ± 2.68	36.46 ± 2.61	25.37 ± 3.02	36.79 ± 2.52	27.17 ± 3.73
	SSIM	0.97 ± 0.01	0.73 ± 0.07	0.98 ± 0.01	0.82 ± 0.05	0.98 ± 0.01	0.90 ± 0.03
	LPIPS	0.41 ± 0.03	0.59 ± 0.03	0.41 ± 0.03	0.55 ± 0.03	0.39 ± 0.03	0.51 ± 0.03
850 nm	PSNR	35.90 ± 2.76	22.08 ± 2.67	36.63 ± 2.58	25.37 ± 3.05	36.94 ± 2.42	27.27 ± 3.81
	SSIM	0.98 ± 0.01	0.74 ± 0.06	0.98 ± 0.01	0.83 ± 0.05	0.98 ± 0.01	0.90 ± 0.03
	LPIPS	0.40 ± 0.03	0.59 ± 0.03	0.41 ± 0.03	0.55 ± 0.03	0.39 ± 0.03	0.51 ± 0.03
920 nm	PSNR	35.64 ± 2.80	21.90 ± 2.55	36.50 ± 2.56	25.17 ± 3.08	36.82 ± 2.41	27.24 ± 3.84
	SSIM	0.98 ± 0.01	0.73 ± 0.06	0.98 ± 0.01	0.82 ± 0.05	0.98 ± 0.01	0.90 ± 0.03
	LPIPS	0.40 ± 0.04	0.59 ± 0.03	0.41 ± 0.04	0.55 ± 0.03	0.29 ± 0.03	0.51 ± 0.03
1,000 nm	PSNR	34.73 ± 2.86	22.10 ± 2.08	35.31 ± 2.45	25.93 ± 2.50	36.16 ± 2.45	27.71 ± 2.84
	SSIM	0.98 ± 0.01	0.80 ± 0.04	0.98 ± 0.01	0.88 ± 0.03	0.98 ± 0.01	0.93 ± 0.03
	LPIPS	0.36 ± 0.03	0.58 ± 0.02	0.37 ± 0.02	0.53 ± 0.03	0.35 ± 0.02	0.47 ± 0.03
1,030 nm	PSNR	35.00 ± 2.81	22.46 ± 2.41	35.85 ± 2.53	26.11 ± 3.06	36.66 ± 2.29	28.31 ± 3.72
	SSIM	0.98 ± 0.01	0.79 ± 0.04	0.98 ± 0.01	0.86 ± 0.03	0.98 ± 0.01	0.92 ± 0.02
	LPIPS	0.37 ± 0.04	0.59 ± 0.03	0.38 ± 0.03	0.54 ± 0.03	0.36 ± 0.03	0.49 ± 0.03
1,064 nm	PSNR	35.08 ± 2.82	22.35 ± 2.51	35.91 ± 2.56	25.93 ± 3.28	36.48 ± 2.31	28.16 ± 3.95
	SSIM	0.98 ± 0.01	0.78 ± 0.04	0.98 ± 0.01	0.86 ± 0.04	0.98 ± 0.01	0.92 ± 0.02
	LPIPS	0.38 ± 0.04	0.59 ± 0.03	0.39 ± 0.03	0.54 ± 0.03	0.36 ± 0.03	0.49 ± 0.03
1,100 nm	PSNR	34.68 ± 2.71	23.25 ± 2.10	35.33 ± 2.31	26.99 ± 2.72	36.02 ± 2.48	29.03 ± 3.18
	SSIM	0.98 ± 0.01	0.82 ± 0.04	0.98 ± 0.01	0.89 ± 0.03	0.98 ± 0.01	0.94 ± 0.01
	LPIPS	0.36 ± 0.03	0.57 ± 0.02	0.38 ± 0.03	0.53 ± 0.02	0.36 ± 0.02	0.47 ± 0.03

### Functional quantification and spectral unmixing

To evaluate the recovery of functional features, we assessed wavelength-dependent optical contrast using quantitative spectral unmixing. Linear unmixing was applied to extract the spatial distributions of deoxygenated hemoglobin (Hb), oxygenated hemoglobin (HbO₂), and total hemoglobin (HbT). Under sparse-sampling conditions (e.g., 32-detector configuration), aliasing artifacts in raw reconstructions propagate across wavelengths, affecting the accuracy of functional maps. The application of OA-UDNet mitigates these artifacts, yielding functional distributions that align more consistently with the 256-detector reference across the evaluated anatomical regions (brain, abdomen, hindlimb, and tumor models; Figs. S11 to S14). Quantitatively, the unmixing errors were reduced. As detailed in Table S6, under the 32-detector configuration, the normalized root mean square error (NRMSE) for HbO₂ and HbT decreased from 7.97% and 8.85% in raw reconstructions to 1.56% and 1.58%, respectively. These results indicate that the framework preserves optical contrast to support functional MSOT assessments.

### Zero-shot generalization on in vivo human datasets

We tested the generalization of OA-UDNet on human data. The model was trained only on small-animal datasets. We directly applied it to in vivo human OAI data acquired with a linear array system.

**Fig. 7.**
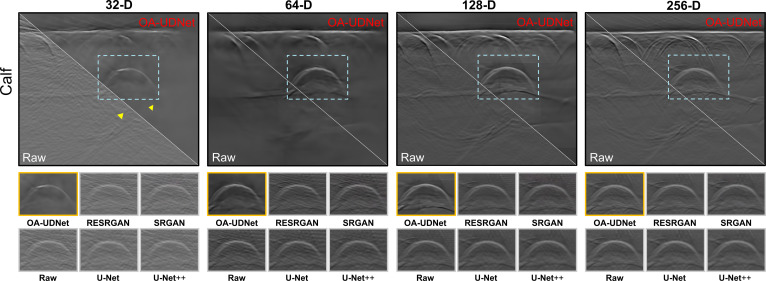
Validation of OA-UDNet on human OAI. Comparisons are presented between OA-UDNet-enhanced OAI, raw OAI, and the enhanced versions using Real-ESRGAN, SRGAN, U-Net, and U-Net++. Yellow boxes highlight notable improvements in image detail and contrast.

Despite clear differences in tissue properties, scale, and device geometry, OA-UDNet showed strong zero-shot performance. In the human calf example (Fig. 7), the raw sparse-view images contained strong acoustic clutter and poor penetration. OA-UDNet reduced these artifacts (yellow arrows) and improved the contrast and continuity of vessels and fascia. To substantiate this cross-species generalizability, we performed a quantitative evaluation across the available human testing cohort (*N* = 44 distinct cross-sectional planes). Full-reference similarity metrics (PSNR and SSIM) were calculated for the sparse configurations using the 256-detector data as the reference. As summarized in Table S7, quantitative analysis demonstrates that OA-UDNet consistently improves reconstruction fidelity over the raw sparse baseline under zero-shot clinical deployments. Similar visual and quantitative trends were consistently observed across the carotid, biceps, and abdominal cross-sections (Fig. S15).

### Quantitative benchmarking against recent methods

To evaluate the performance of OA-UDNet against recent approaches, we conducted a quantitative comparison with 3 photoacoustic computed tomography sparse-reconstruction methods published between 2024 and 2026 that provided open-source codebases: AD-WaveNet [18], PA OmniNet [19], and PAT-ADN [20]. To ensure a consistent comparison, all competing frameworks were retrained from scratch using our training dataset under the 32-detector sparse configuration and evaluated on the same independent test set. As summarized in Table 3, under this sparse-sampling condition, the baseline models yielded PSNR values ranging from 23.15 to 32.33 dB. In comparison, OA-UDNet demonstrated higher performance across the evaluation metrics, achieving a PSNR of up to 38.99 dB (in the brain region) and an SSIM of 0.983. These results indicate that OA-UDNet improves reconstruction accuracy and structural fidelity compared to the evaluated methods under sparse conditions.

**Table 3.** Quantitative performance benchmarking against recent SOTA

Datasets	Metrics	AD-WaveNet	PA_OmniNet	PAT-ADN	OA-UDNet
Abdomen	PSNR	26.17 ± 1.55	32.33 ± 3.74	27.96 ± 2.36	36.56 ± 2.11
	SSIM	0.70 ± 0.03	0.97 ± 0.02	0.89 ± 0.02	0.979 ± 0.005
Brain	PSNR	25.30 ± 2.39	31.84 ± 4.31	26.32 ± 3.09	38.99 ± 1.91
	SSIM	0.63 ± 0.05	0.97 ± 0.03	0.86 ± 0.02	0.983 ± 0.004
Hindlimb	PSNR	23.15 ± 3.73	30.82 ± 4.79	26.28 ± 3.33	31.95 ± 4.88
	SSIM	0.62 ± 0.06	0.96 ± 0.03	0.84 ± 0.05	0.965 ± 0.018
Tumor	PSNR	24.56 ± 2.91	31.54 ± 4.67	27.14 ± 3.10	36.31 ± 2.26
	SSIM	0.65 ± 0.05	0.97 ± 0.02	0.85 ± 0.04	0.976 ± 0.009

### Ablation studies on network architecture

We performed ablation studies on the 32-detector mouse dataset to validate our design. We focused on the EAS module and the number of diffusion steps. We trained a baseline model without the EAS module (w/o EAS). As detailed in Table 4, removing the EAS module markedly degraded reconstruction fidelity, resulting in a PSNR drop of 3.91 dB, a reduction in SSIM from 0.975 to 0.969, and a substantial degradation in perceptual quality, with the LPIPS worsening to 0.514 ± 0.057. Without the deterministic Sobel prior, the network could not reliably separate true structural boundaries from background noise. This result shows that the EAS module is important for preserving structural details in noisy data.

**Table 4.** Ablation study on the edge-aware self-attention (EAS) module using the 32-detector mouse dataset

Model configuration	PSNR (dB) ↑	SSIM ↑	LPIPS↓
OA-UDNet (w/o EAS)	31.480 ± 5.420	0.969 ± 0.019	0.514 ± 0.057
OA-UDNet (full)	35.390 ± 0.440	0.975 ± 0.005	0.437 ± 0.035

Standard diffusion models use many steps and are slow. OA-UDNet uses only 10 steps (*T* = 10). We tested 1, 5, 10, and 20 steps (Fig. S16). Increasing to 20 steps provided negligible gain (less than 0.1 dB in PSNR, with minimal change in SSIM) but doubled the computational overhead (Fig. S16). Furthermore, we benchmarked our short-chain process against a standard denoising diffusion probabilistic model (DDPM) utilizing a large number of steps (e.g., 1,000 steps) from pure Gaussian noise without an image-space prior. The standard DDPM struggled severely with the extreme sparsity, yielding significantly lower PSNRs (detailed in Table S8) while requiring approximately 15,000 ms per frame on a single NVIDIA RTX 4090 GPU. In stark contrast, our preconditioned short-chain OA-UDNet achieves superior structural accuracy with an inference time of only ~150 ms per frame. Therefore, the proposed short-chain configuration provides an optimal and clinically viable balance between generative accuracy and absolute inference speed.

## Discussion

Despite recent advances in optoacoustic system design and data processing that have substantially increased imaging frame rates [21], the intrinsic unidirectional propagation of ultrasound in biological tissues remains a fundamental constraint of OAI [22]. While sparse data acquisition accelerates imaging and reduces hardware costs, it severely degrades spatial resolution and diagnostic sensitivity, leading to severe ill-posed reconstruction problems [23,24]. To overcome this, we developed OA-UDNet, a tailored hybrid dual-space diffusion framework. OA-UDNet successfully reconstructs highly undersampled 32-detector data, achieving high structural fidelity (e.g., average SSIM of 0.976) relative to conventional 256-detector references. This approach mitigates current hardware constraints, providing a clear computational pathway for low-cost, handheld clinical optoacoustic devices [25].

Previous methods often treat denoising and image restoration as separate tasks [26]. However, under extreme sparse-view conditions, aliasing artifacts and loss of high-frequency details occur together. Sequential processing leads to error accumulation and structural distortion [27]. OA-UDNet addresses this problem with a different strategy. It first uses an encoder–decoder to build a stable and dense image-space representation. This step restores the main structure and low-frequency content. The following diffusion process does not need to model the global background. It focuses on refining high-frequency details. This joint design improves performance. It avoids the limitations of standard regression models such as U-Net and U-Net++.

This architectural design directly justifies our highly accelerated, 10-step short-chain diffusion process. Standard diffusion models demand hundreds to thousands of iterative steps [28], rendering them computationally prohibitive for real-time medical imaging [29]. Our preconditioned image-space projection drastically truncates this chain, achieving an optimal algorithmic balance between generative precision and inference speed. Furthermore, unconstrained attention mechanisms in extremely low-SNR scenarios are highly susceptible to overfitting to background noise due to a lack of inductive biases [30]. By embedding a fixed, deterministic Sobel operator within the EAS module, we force the network to align strictly with macroscopic anatomical boundaries. This “hard-coded” geometric prior stabilizes the generation process [31], preserving diagnostic edge details across highly heterogeneous tissues without hallucinating spurious artifacts. In in vivo reconstructions, 32-detector images occasionally exhibit a more textured background than 64-detector results. Under severe signal depletion, the network’s effort to restore high-frequency boundaries inadvertently amplifies residual aliasing noise. These visual textures represent noise artifacts, not hallucinated anatomical structures. Increased physical acoustic information (e.g., 64 detectors) naturally enables smoother convergence and a cleaner background.

The superiority of this framework is clearly reflected in our quantitative benchmarks against recent state-of-the-art (SOTA) methodologies. Existing advanced models struggle substantially under extreme sparsity. For instance, in the highly undersampled 32-detector setting, recent transformer-based and regression models such as PACformer [32], TT-PADM [33], IR-SDE [34], DM [10], PA OmniNet [19],and PAT-ADN [20] yield PSNR values of only 28.13 to ~30 dB, respectively. In contrast, OA-UDNet attains a PSNR of 35.95 dB and an SSIM of 0.976 under identical conditions. This substantial margin of over 7 dB highlights the superior robustness of OA-UDNet compared to the evaluated contemporary methods for sparse-view OAI.

While early deep learning approaches for sparse-view optoacoustic reconstruction (e.g., standard U-Net) successfully suppressed artifacts, their deterministic architectures often cause oversmoothing and loss of fine vasculature under extreme sparsity (e.g., 32 detectors). In contrast, OA-UDNet employs a hybrid dual-space generative framework that combines an encoder–decoder with a highly accelerated 10-step short-chain diffusion process. This design restores high-frequency boundaries while using an edge-aware prior (via the EAS module) to prevent structural hallucinations. Consequently, OA-UDNet achieves substantial quantitative gains over standard U-Nets. It improves PSNR by over 12 dB and yields a near-ideal SSIM (>0.96) under a 32-detector configuration, representing a substantial advance in structural and functional reconstruction fidelity.

Despite these strong performances and the demonstrated zero-shot generalizability on human clinical data, this study has limitations. Although the model demonstrated zero-shot generalizability to human line-array data, the training corpus was predominantly composed of in vivo murine models. Expanding the training manifold with large-scale, diverse clinical human datasets will be essential for future clinical translation. Additionally, while our in vivo results show that OA-UDNet considerably reduces functional spectral unmixing errors (NRMSE < 1.6%), absolute oxygen-saturation calibration in dynamic physical phantoms was not performed. Future work will include dynamic phantom validations to further assess the accuracy of quantitative functional parameter estimation under varying oxygenation levels. Finally, future work will focus on optimizing the network for deployment on edge-computing devices to evaluate its real-time inference latency in live clinical workflows precisely.

## Conclusion

OA-UDNet establishes a new benchmark for sparse-sampling MSOT. By integrating a short-chain diffusion process with a fixed edge-aware geometric prior, the method effectively circumvents the structural distortion of conventional regression models and the computational latency of prolonged generative diffusion. OA-UDNet consistently achieves massive quantitative margins over existing SOTA methods under extreme sparsity and demonstrates robust generalizability across diverse murine organs, multispectral wavelengths, and human clinical datasets. This framework considerably alleviates the hardware density requirements of current optoacoustic systems, offering a highly reliable and cost-effective computational pathway toward the clinical translation of high-fidelity functional imaging.

## Materials and Methods

### Data acquisition and preprocessing

Photoacoustic imaging data were acquired using a MSOT system (inVision ECHO, iThera Medical GmbH) equipped with a multi-wavelength laser (660 to 1,300 nm) emitting short pulses (<10 ns) at 20 Hz. The system used a ring-shaped ultrasound transducer array (256 elements, 4 cm radius, 310° angular coverage, center frequency 5 MHz, >60% bandwidth in transmit/receive mode). To independently validate structural fidelity and acoustic modeling, 4 physical phantoms with well-defined discrete geometries were imaged. Animals were positioned in a specialized holder to align with the transducer, and purified water was used for acoustic coupling. Whole-body scans were performed on 7-week-old male athymic nude mice at step sizes of 0.5 mm, collecting multispectral data at 12 wavelengths (680 to 1,100 nm). Tumor imaging involved orthotopic injection of ~1 × 10^6^ tumor cells into the tibiae of 6- to 7-week-old nude mice. During imaging, anesthesia was maintained using 2% isoflurane, and body temperature was controlled at 36°C. Sparse-sampling datasets were generated by simulating uniformly spaced detector arrays at down-sampling ratios of 1:2, 1:4, and 1:8, corresponding to 128, 64, and 32 detectors. Human data were obtained from a public dataset with 44 full-view sinograms (256 detectors, 800 nm). All 2-dimensional (2D) cross-sectional images were reconstructed into 332 × 332 matrices using a standard back-projection (BP) algorithm [35], after applying band-pass filters to reduce noise and remove low-frequency offsets (Fig 1A). For more experimental details, including anesthesia protocol, animal handling, tumor injection, and imaging parameters, see Note S4.

### Framework overview

We propose OA-UDNet, a hybrid dual-space refinement framework for extreme sparse-view OAI (Fig. 1B). Unlike conventional methods that treat denoising and image restoration as separate steps, OA-UDNet performs both tasks in a single pipeline. The entire forward propagation logic is explicitly organized into 3 sequential stages. Stage 1: Feature compression and initial reconstruction. The encoder–decoder maps the degraded sparse input to a bottleneck latent feature and decodes it into a dense initial image-space estimate. Stage 2: Iterative image-space diffusion loop. A short diffusion process with only 10 steps is applied directly to this reconstructed image tensor in the spatial domain. A dedicated EUNet removes noise step-by-step and recovers high-frequency details. Stage 3: Final generation. The denoised image is passed through the shared encoder–decoder one final time to enforce structural consistency, generating the final high-fidelity restored output. This design combines the generative strength of diffusion models with the stability of deterministic autoencoders. It reduces inference time and avoids the strong structural distortions often seen in highly sparse MSOT reconstruction.

### Short-chain forward diffusion process

To systematically model the noise and aliasing artifacts in sparse MSOT images, we introduce a forward diffusion process ${\left\{x_{t}\right\}}_{t=0}^{T}$. Given the initial image-space estimate $x_{0}$, the forward process gradually adds Gaussian noise according to a variance schedule:

$$x_{t}=\sqrt{\alpha_{t}}x_{t-1}+\sqrt{1-\alpha_{t}}\epsilon_{t},\epsilon_{t}∼N\left(0,\;I\right)$$ (1)

$${\bar{\alpha }}_{t}=\prod_{k=1}^{t}\;\left(1-\beta_{k}\right)$$ (2)

where $\beta_{t}\in \left(0,1\right)$ dictates the noise variance at step *t*. Unlike standard DDPMs [29] that start from pure Gaussian noise, our framework leverages the structural prior established by the initial encoder–decoder. Consequently, we employ a linear noise scheduling strategy (e.g., β linearly increasing from 0.1 to 0.2) and drastically truncate the total diffusion timesteps to *T* = 10. This “short-chain” diffusion design is highly intentional: The initial encoder–decoder effectively resolves low-frequency structural components, allowing the diffusion process to focus exclusively on refining high-frequency residuals and complex noise distributions. This reduces the computational burden by orders of magnitude while avoiding the oversmoothing effects associated with long-chain Markov transitions.

### Reverse diffusion with EUNet

The reverse process estimates stepwise noise to recover the refined image. The reverse transition is parameterized as:

$$p_{\theta }\left(x_{t-1}|x_{t}\right)=N\left(x_{t-1};{\;\mu }_{\theta }\left(x_{t},\;t\right),{\;\beta }_{t}\Sigma_{t}\right),$$ (3)

$$\mu_{\theta }\left(x_{t},\;t\right)=\frac{1}{\sqrt{\alpha_{t}}}\left(x_{t}-\frac{\beta_{t}}{\sqrt{1-\alpha_{t}}}\epsilon_{\theta }\left(x_{t},\;t\right)\right)$$ (4)

where $\epsilon_{\theta }\left(X_{t},\;t\right)$ is the noise predicted by our customized EUNet. During the 10 iterative steps, the EUNet leverages spatial attention to progressively enhance boundaries and suppress artifacts, achieving collaborative optimization of denoising and high-fidelity image restoration.

### EAS module

A critical challenge in extreme sparse-view MSOT is that standard attention mechanisms often overfit to severe background noise, introducing spurious high-frequency artifacts. To mitigate this, we introduce the EAS module, which embeds a deterministic geometric prior into the network.

The EAS module employs a localization network $N_{\mathrm{loc}}$ (comprising convolutional and max-pooling layers) to extract coarse feature responses from the input 𝑥. Instead of learning unconstrained edge filters, we explicitly apply fixed Sobel convolution operators to these localized features:

E=Sx∗z2+Sy∗z2 (5)

where ∗ denotes the convolution operation, and 𝑆𝑥 and 𝑆𝑦 are the horizontal and vertical Sobel kernels, respectively. Crucially, the weights of 𝑆𝑥 and 𝑆𝑦 are strictly fixed (no gradients are computed during training). By freezing these kernels, we prevent the network from memorizing noise patterns, ensuring that the edge prior remains robust even under realistic, high-noise conditions. The resulting edge map *E* is then flattened and passed through fully connected layers to predict an affine transformation matrix $:\theta \in {\mathbb{R}}^{2\times 3}$:

$$\theta =\mathrm{FC}\left(E\right)$$ (6)

Finally, a differentiable grid sampling operation generates an affine grid $\Gamma_{\theta }\left(G\right)$ based on θ and applies this transformation to the original input feature map x:

$$x^{\prime }={\text{grid}}_{\text{sample}}\left(x,{\;\Gamma }_{\theta }\left(G\right)\right)$$ (7)

This explicitly enforces spatial alignment and boundary preservation across the EUNet layers during the reverse diffusion process.

### Network architecture details

To ensure reproducibility, the detailed layer configurations are as follows: The encoder consists of 4 sequential Conv2d blocks (kernel size 4, stride 2, padding 1/2), each followed by a ReLU activation, mapping the 1-channel input to a 512-channel latent representation. The decoder includes 4 ConvTranspose2d blocks (kernel size 4, stride 2, padding 1/2) with ReLU activations to restore spatial resolution, and the final layer uses a Sigmoid activation to map the output to the range (0, 1). Specifically, this architecture performs an explicit 4× spatial upscaling mapping from the low-density input matrix to computationally recover high-frequency structural boundaries. EUNet follows a symmetric 4-level U-Net structure, where each encoding block contains an EAS module and two 3 × 3 convolutional layers, with downsampling performed via 2 × 2 max-pooling. The decoder uses transposed convolutions for upsampling and incorporates skip connections, fusing features through center cropping and concatenation to integrate multi-scale information.

### Model training and objective function

It is important to note that OA-UDNet operates under 2 distinct training paradigms depending on the target task. (a) Supervised sparse-view reconstruction: For inputs from 32-, 64-, and 128-detector arrays, the network is trained in a supervised manner, utilizing the corresponding 256-detector data as the high-resolution ground-truth target. (b) Unsupervised identity mapping: To process the full-view 256-detector data itself, where a higher-density physical ground truth is unavailable, the model undergoes unsupervised training (where the 256-detector data serve as both input and target) to suppress residual systemic hardware-induced noise without relying on external references.

While 256-detector data are conventionally treated as the “ground truth”, it intrinsically retains minor systemic noise and subtle artifacts. By training the network to reconstruct the 256-detector data through our edge-aware diffusion pipeline, the model learns a robust, noise-free target data manifold. Consequently, by passing the raw dense-array data through our edge-aware diffusion pipeline in this unsupervised, autoencoder-like manner, the network learns to suppress residual systemic hardware-induced noise without relying on external higher-density references.

For each detector configuration, 7,000 images were set aside as the test set, while the remaining 64,400 images were split into training and validation sets at an 8:2 ratio. The model was trained for 100 epochs using the Adam optimizer with a batch size of 16 and a learning rate of 1 × 10^−4^. Because OA-UDNet wraps the diffusion process within an encoder–decoder pipeline, the entire framework is trained end-to-end. We optimize the network using a global mean squared error (MSE) loss between the final output and the 256-detector ground truth:

$$L_{\mathrm{MSE}}=\frac{1}{N}\sum_{i=1}^{N}\;{∥x_{\mathrm{hr}}^{\left(i\right)}-x_{\text{final}}^{\left(i\right)}∥}_{2}^{2}$$ (8)

Unlike standard DDPMs that compute loss solely on the added noise ε, our end-to-end MSE formulation directly penalizes structural deviations in the final reconstructed image space. This ensures maximum pixel-level fidelity and simplifies the optimization landscape for joint denoising and image restoration.

### Comparison of baseline methods

To validate the superiority of OA-UDNet, we conducted a quantitative comparison with SOTA methods for image denoising and restoration across 3 distinct datasets: an animal imaging dataset and a human imaging dataset. Each dataset comprised paired full-view (256-detector) and sparse-sampling images. The methods used for comparison were Real-ESRGAN [36], SRGAN [37], U-Net [38], and U-Net++ [39]. For a fair comparison, these models were retrained on the same denoising and image restoration training sets following standard procedures and optimized parameters as reported in the relevant studies [12]. The enhanced images were rescaled to match the 256-detector reference using nearest-neighbor interpolation, enabling the computation of quantitative evaluation metrics.

### Performance metrics

To rigorously evaluate OA-UDNet performance, we employed a set of objective metrics tailored to detector density and availability of ground-truth references. For sparse-array configurations (32, 64, and 128 detectors), we computed the LPIPS, which assesses perceptual similarity by calculating distances between multi-scale feature maps extracted from a pretrained VGG network and aggregated using learned weights. Performance metrics were strictly assigned based on the operational task. For the supervised sparse-view reconstructions (32, 64, and 128 detectors), full-reference metrics, specifically PSNR and SSIM, were calculated using the 256-detector images as the absolute ground truth. Conversely, for the unsupervised identity mapping task on the 256-detector data, the absence of a higher-density reference makes full-reference computation mathematically impossible. Consequently, 2 no-reference metrics, average local contrast (ALC) and energy of gradient (EOG), were employed to quantify the network’s inherent noise-suppression characteristics (Notes S5 and S6). All metrics were computed on an independent test set, and comparisons with baseline methods were performed using 2-sided *t* tests, with *P* < 0.05 considered statistically significant.

## Data Availability

Data will be made available on request.

## Supplementary Materials

Supplementary 1 Figs. S1 to S16 Tables S1 to S8 Notes S1 to S6
