# Supplementary material for: Edge-Aware Short-Chain Diffusion Enables High-Fidelity Sparse-Sampling Optoacoustic Tomography
Source: BME Front. 2026 Jul 23;7:0292. doi: 10.34133/bmef.0292 (PMC13392286; doi:10.34133/bmef.0292)
Supplement: Supplementary 1 — Figs. S1 to S16 Tables S1 to S8 Notes S1 to S6 [file bmef.0292.f1.docx]

**Supplementary materials for:**

**Edge-aware short-chain diffusion enables high-fidelity sparse-sampling optoacoustic tomography**

**Figs. S1 to S16**

**Notes 1 to 6**

**Table S1-S8**


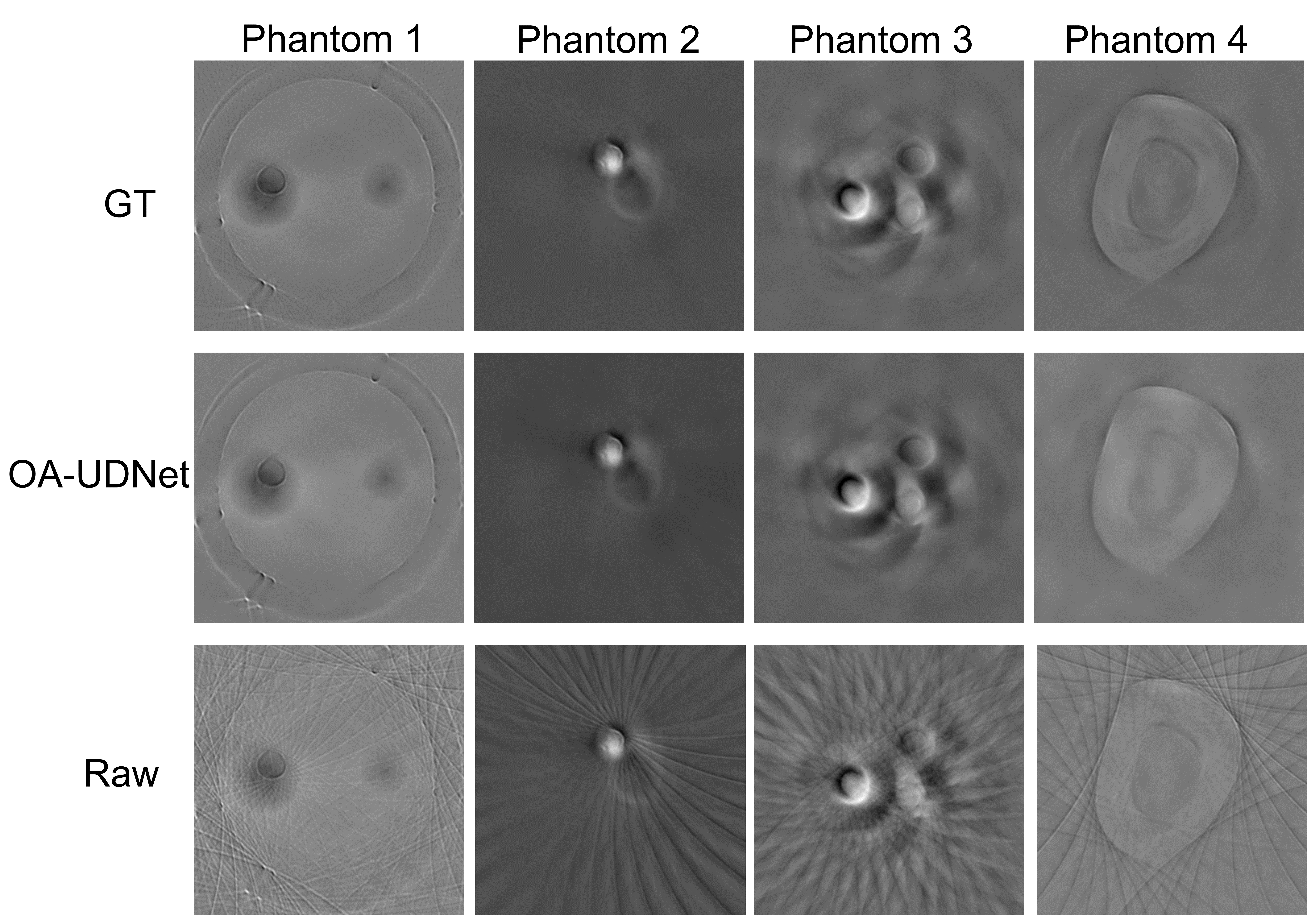
 **Supplementary Fig. S1**. Validation of structural fidelity using a customized phantom. Visual comparison of the Ground Truth (GT), the OA-UDNet enhanced images, and the raw sparse reconstructions across four distinct target geometries (Phantom 1–4) under the extremely sparse 32-detector sampling configuration.

**Supplementary Table S1**. Quantitative performance comparison on the physical phantom dataset under 32-detector sparse sampling configurations.

| Model | PSNR | SSIM | LPIPS |
| --- | --- | --- | --- |
| OA-UDNet | 37.3471±3.4219 | 0.9859±0.0106 | 0.3628±0.0606 |
| Raw | 26.5226±2.1663 | 0.6807±0.0835 | 0.4568±0.0225 |

**Result section**

Supplementary Note 1**: Quantitative Evaluation of OA-UDNet for Mice Brain OAI Using 64-,128-, 256 detectors**

For the 64-detector array data, PSNR improved from 26.26 ± 2.86 dB to 38.68 ± 2.0 dB (38.99 ± 22.98%), SSIM increased from 0.83 ± 0.05 to 0.983 ± 0.0044 (19.06 ± 6.74%), and LPIPS decreased from 0.55 ± 0.02 to 0.42 ± 0.02 (24.03 ± 2.50%). Similarly, for the 128-detector array data, PSNR rose from 28.34 ± 3.85 dB to 39.22 ± 1.56 dB (41.17 ± 21.52%), SSIM improved from 0.90 ± 0.03 to 0.9844 ± 0.0035 (9.45 ± 3.73%), and LPIPS declined from 0.51 ± 0.02 to 0.39 ± 0.02 (22.24 ± 2.51%). For the 256-detector array data, OA-UDNet also demonstrated superior performance, with ALC increasing from 0.64 ± 0.13% to 0.74 ± 0.18% (14.33 ± 9.34%) and EOG rising from 4.22 ± 0.97 to 4.89 ± 1.27 (15.27 ± 8.81%).

Supplementary Note 2**:** Quantitative Evaluation of OA-UDNet for Mice Abdominal OAI Using 64-,128-, 256 detectors

For datasets obtained with 64 detectors, PSNR improved from 28.16±1.94 dB to 36.64±2.04 dB (30.79 ± 12.00% increase), SSIM rose from 0.88±0.04 to 0.98±0.01 (11.36 ± 5.04% increase), and LPIPS decreased from 0.52±0.03 to 0.35±0.03 (31.87 ± 4.17% decrease). For the 128-detector data, PSNR improved from 30.3±2.26 dB to 36.94±2.02 dB, reflecting a 22.60 ± 11.17% increase, SSIM increased from 0.93±0.02 to 0.98±0.01 (4.98 ± 2.71% improvement), and LPIPS dropped from 0.46±0.03 to 0.34±0.03 (26.34 ± 3.52% decrease). For the 256-detector data, additional evaluation metrics, including edge orientation gradient (EOG) and average lightness contrast (ALC), were used. EOG increased from 4.94±0.64% to 6.12±0.97%, corresponding to a 23.91 ± 13.14% improvement, and ALC rose from 0.74±0.12% to 0.87±0.18%, reflecting a 17.12 ± 10.09% gain.

Supplementary Note 3**:** Quantitative Evaluation of OA-UDNet for Hindlimb and Tumor OAI in Mice Using 64-,128- , 256 detectors

For the quantitative evaluation of the hindlimb dataset, the proposed method (OA-UDNet) demonstrated substantial improvements across multiple imaging configurations. In the 64-detector array, the PSNR increased from 22.24±5.31dB to 32.60±2.38 dB (26.58 ± 29.35%), the SSIM improved from 0.81±0.06 to 0.97±0.01 (20.17 ± 8.18 %), and the LPIPS decreased from 0.6±0.04 to 0.42±0.06 (41.81 ± 28.66 %). Similarly, in the 128-detector array, the PSNR increased from 25.52±4.79 dB to 33.06±4.48 dB (34.88 ± 34.00%), the SSIM improved from 0.89±0.04 to 0.98±0.01 (10.15 ± 5.08 %), and the LPIPS declined from 0.51±0.04 to 0.39±0.05 (34.48 ± 22.32%). Furthermore, in the 256-detector array, OA-UDNet exhibited superior performance, with the ALC increasing from 0.0077±0.002 to 0.0092±0.0029 (18.49 ± 15.79%) and the EOG rising from 4.38±1.12 to 5.4±1.86 (20.89 ± 15.94%).

For the quantitative evaluation of the osteosarcoma dataset, a similar trend of performance enhancement was observed. In the 64-detector array, the PSNR increased from 23.76±3.63 dB to 36.3±2.09 dB (56.84 ± 29.00%), while the SSIM improved from 0.81±0.06 to 0.98±0.01 (20.69 ± 8.97%), and the LPIPS decreased from 0.5584±0.0308 to 0.4232±0.036 (33.16 ± 16.48%). Similarly, in the 128-detector array, the PSNR increased from 25.65±4.21 dB to 36.96±1.98 dB (48.57 ± 29.24%), the SSIM improved from 0.89±0.04 to 0.98±0.01 (10.45 ± 5.17%), and the LPIPS declined from 0.5072±0.0337 to 0.3866±0.0337 (32.42 ± 16.85%). Notably, in the 256-detector array, OA-UDNet continued to outperform baseline methods, with ALC increasing from 0.0075±0.0022 to 0.0091±0.0031% (19.68 ± 11.63%) and EOG rising from 4.96±1.11% to 6.12±1.74% (22.04 ± 12.36%). These results underscore the efficacy of OA-UDNet in enhancing image quality and structural fidelity across different detector configurations in the tumor dataset.


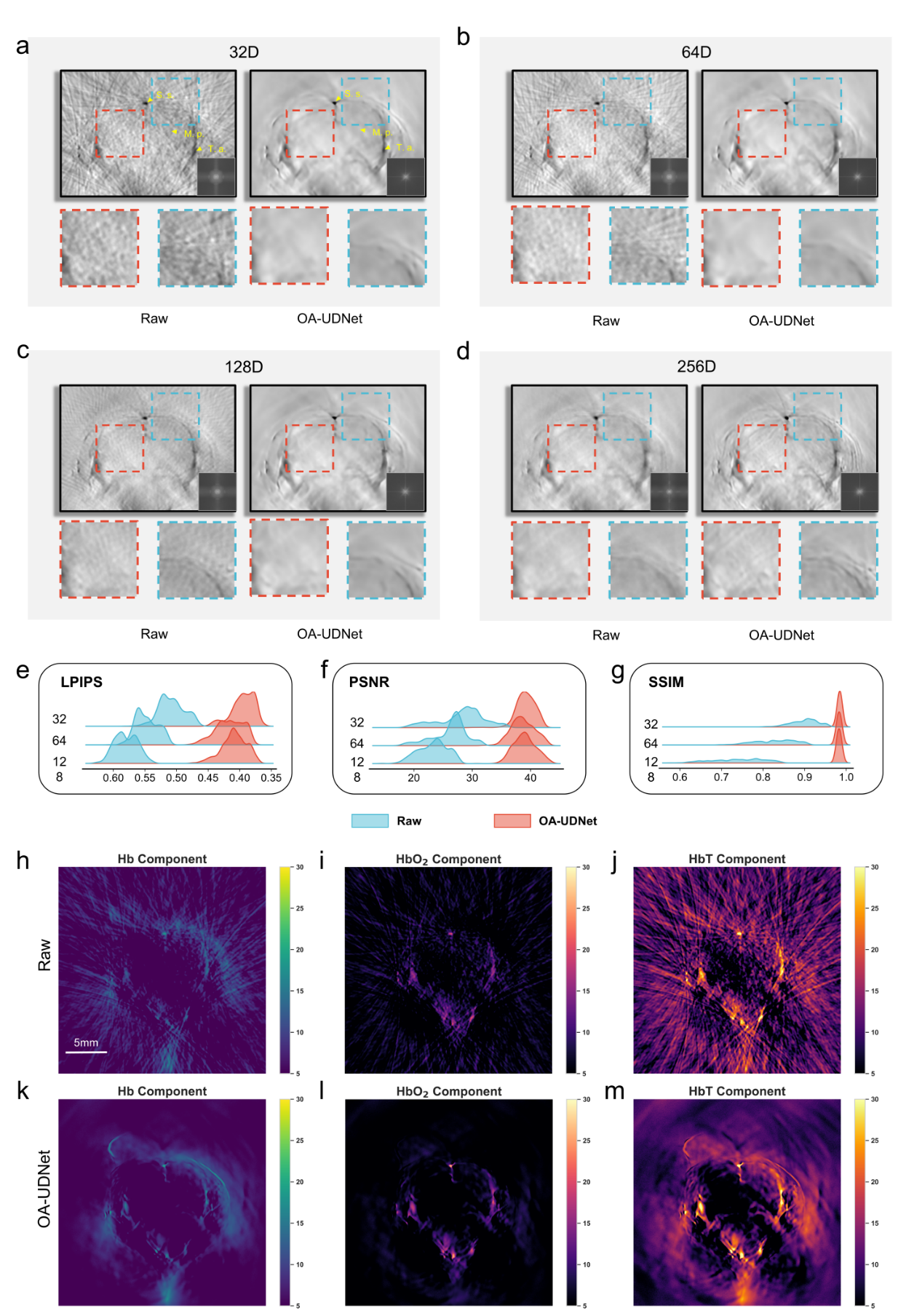


Supplementary Fig.S2. (a–d) Representative OAI of mouse brains acquired with 32-, 64-, 128-, and 256-detector arrays at 800nm. Comparisons are presented between raw OAI (left) and OA-UDNet-enhanced OAI (right). The bottom-right inset displays the corresponding Fourier spectra. The red and blue boxes emphasize local structural details of the brain. S.s. denoting the sagittal sinus, M.p. denoting the mesencephalon, T.a. denoting the temporal artery.


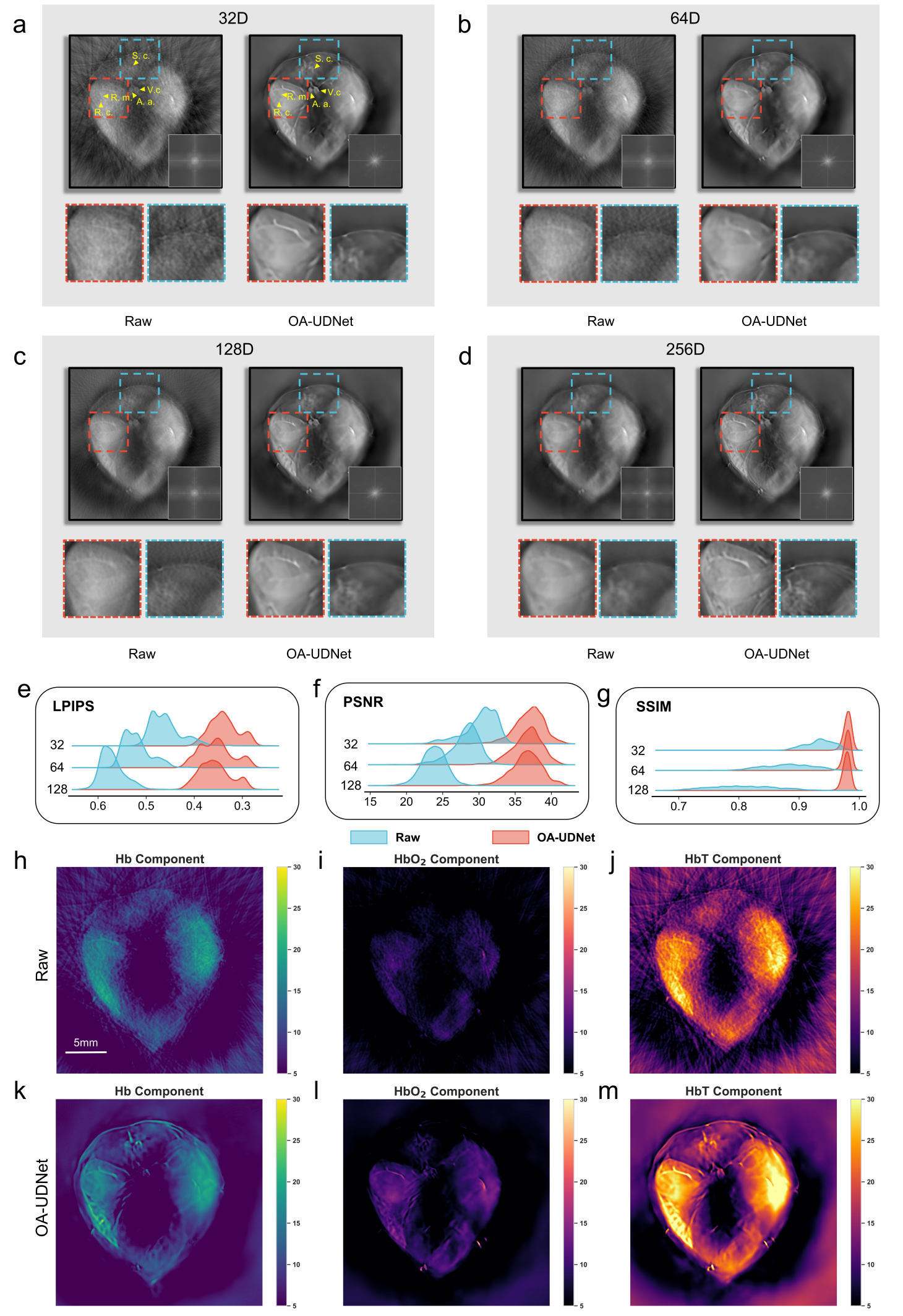


Supplementary Fig.S3. (a–d) Representative optoacoustic images (OAIs) of the mouse abdomen acquired at 800 nm using 32-, 64-, 128-, and 256-detector arrays. For each configuration, the left panel shows the raw OAI and the right panel the OA-UDNet–reconstructed OAI. The bottom-right inset displays the corresponding Fourier spectra. Red and blue boxes highlight local anatomical details: R.m. (renal medulla), R.c. (renal cortex), S.c. (spinal cord), V.c. (vena cava), and A.a. (abdominal aorta).


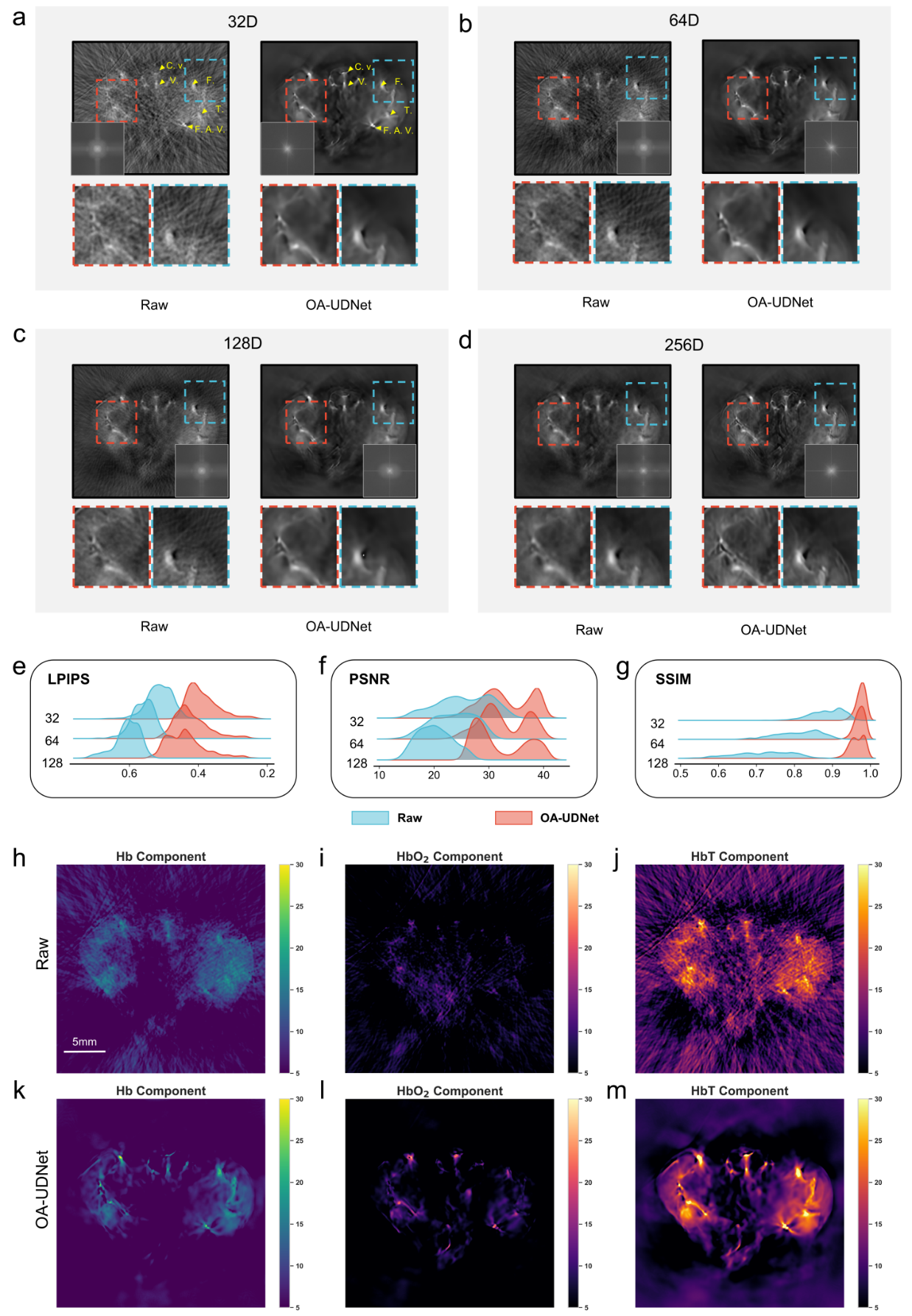


Supplementary Fig. S**4**. (a–d) Representative OAI of the mouse hindlimb acquired with 32-, 64-, 128-, and 256-detector arrays at 800 nm. Comparisons are shown between raw OAI (left) and OA-UDNet-enhanced OAI (right), with red and blue boxes highlighting local structural details. The bottom-right inset displays the corresponding Fourier spectra. C.v., caudal vertebrae; V., vein; F., fibula; T., Hindlimb; F.A.V., femoral artery and vein.


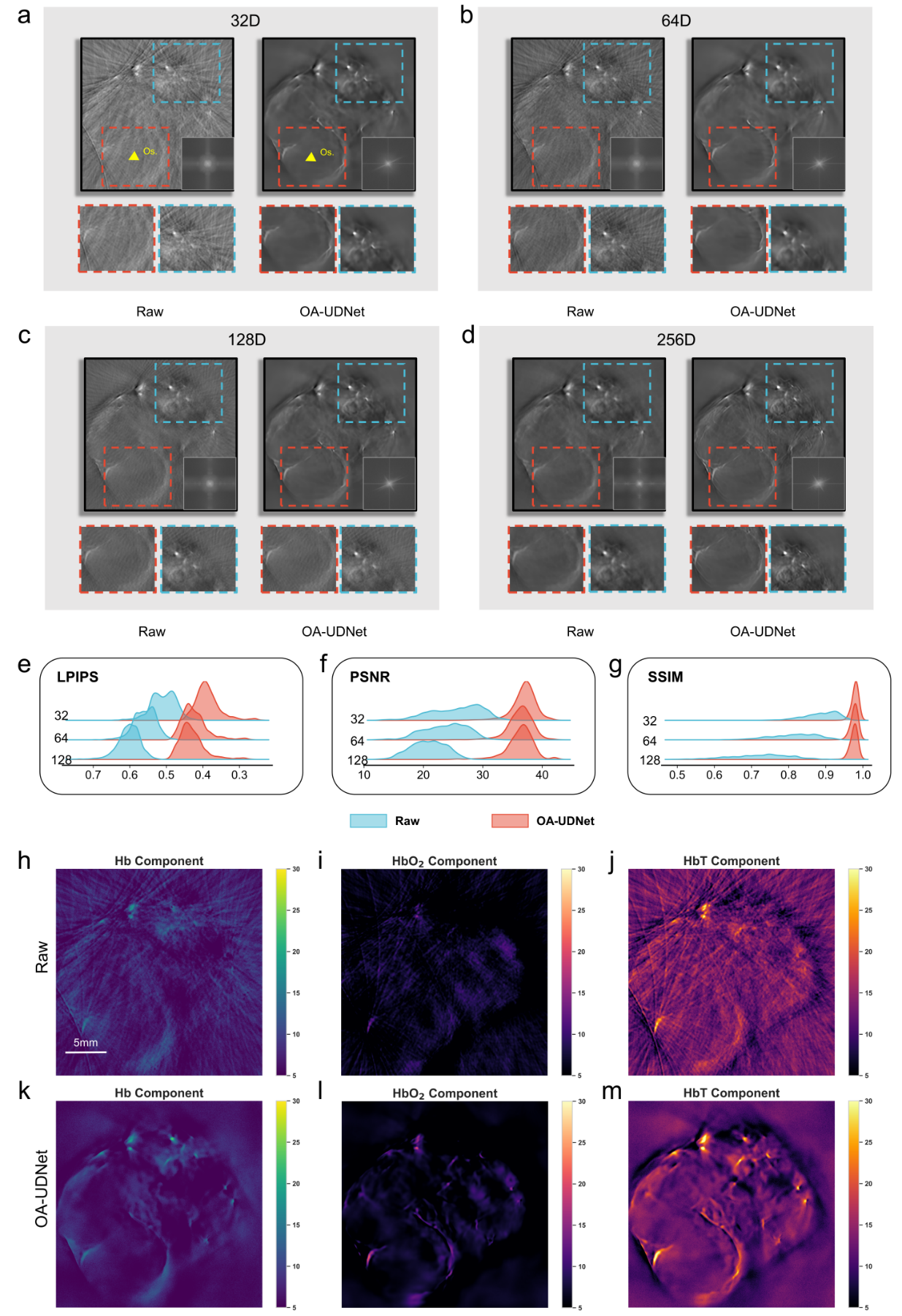


Supplementary Fig. S**5** (a–d) Representative OAI images of mouse osteosarcoma acquired with 32-, 64-, 128-, and 256-detector arrays at 800 nm. The bottom-right inset displays the corresponding Fourier spectra. Comparisons between raw OAI (left) and OA-UDNet-enhanced OAI (right) are shown, with red and blue boxes emphasizing local structural details.


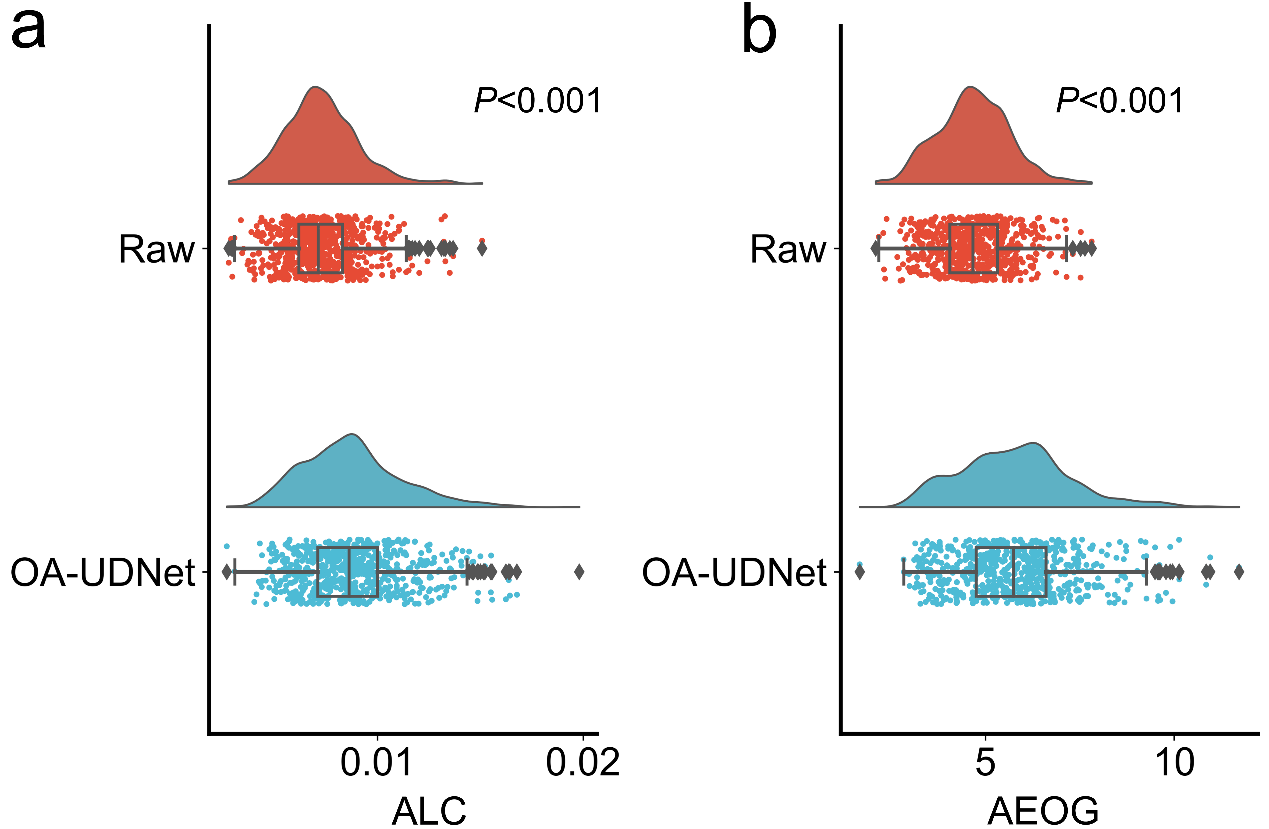


Supplementary Fig.S6. Comparison of average local contrast (ALC) and average energy of gradient (AEOG) scores with raw OAI in a 256-detector array for the mouse test dataset.


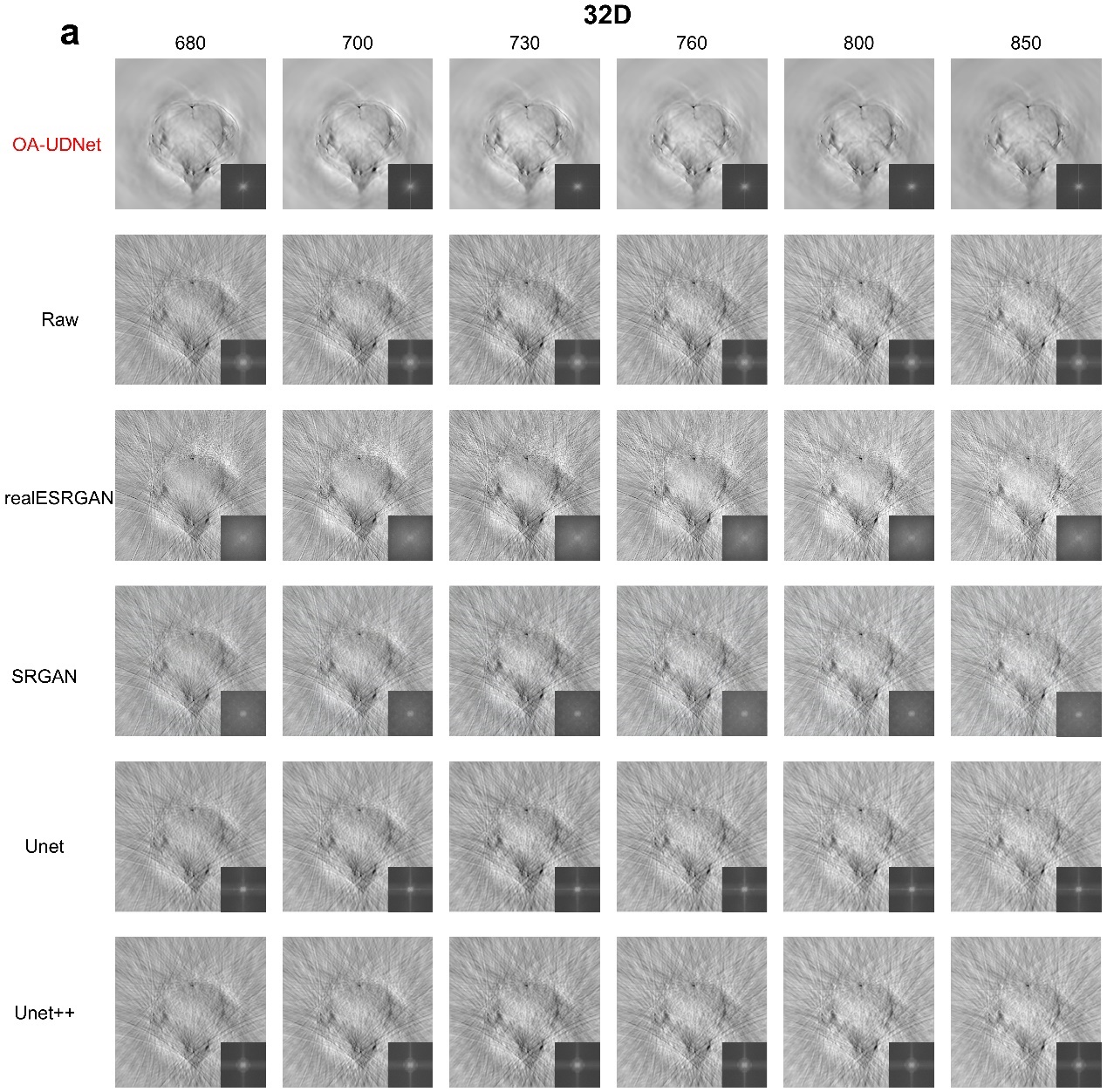


Supplementary Fig.S7: a Visual comparison of optoacoustic imaging (OAI) enhancement in the brain region achieved by OA-UDNet and four methods - Real-ESRGAN, SRGAN, U-Net and U-Net++ over 680nm, 700nm, 730nm, 760nm, 800nm, 850nm in 32-detector arrays data. The Fourier spectrogram is shown in the lower right.


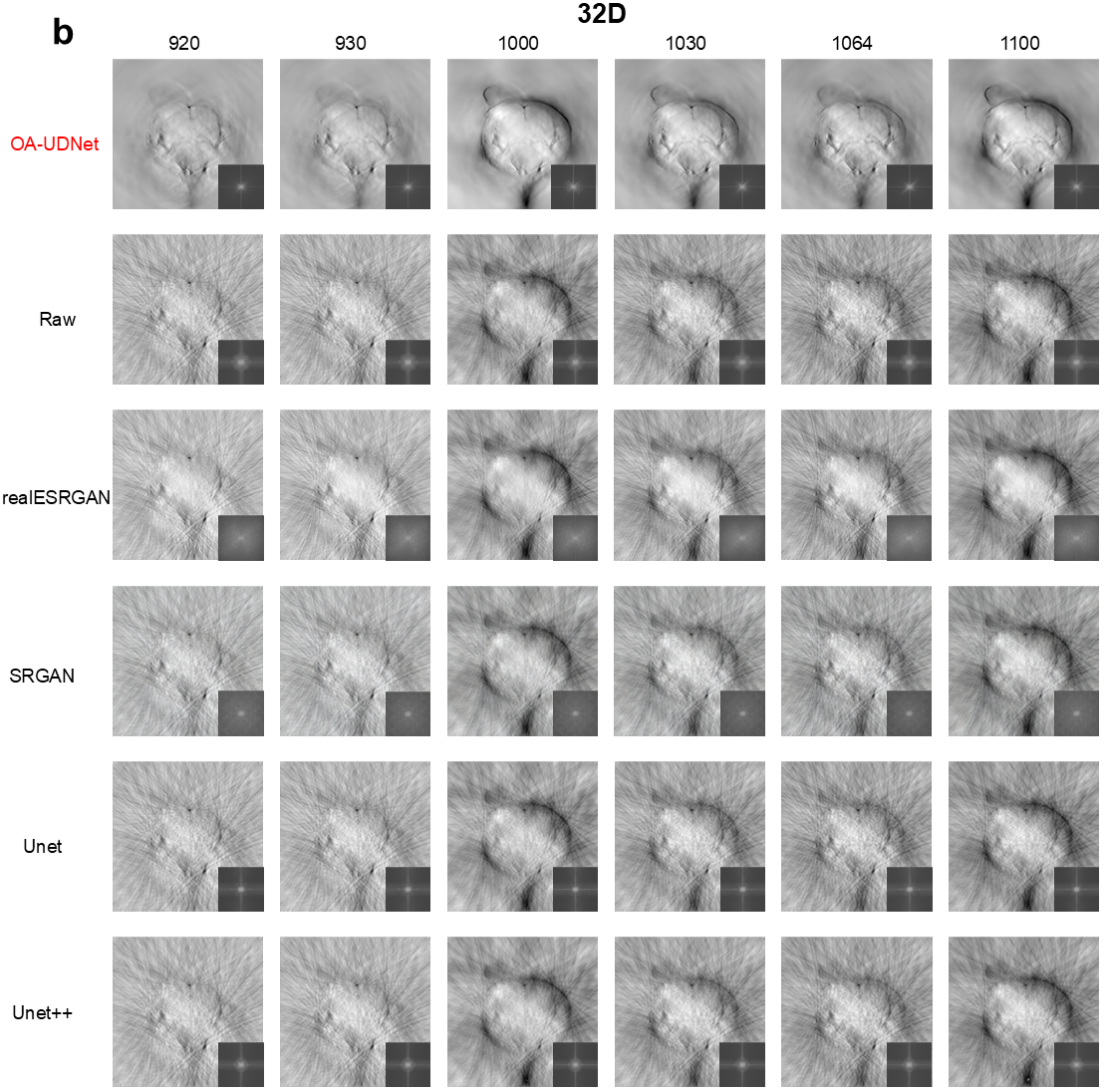


Supplementary Fig.S7: b Visual comparison of optoacoustic imaging (OAI) enhancement in the brain region achieved by OA-UDNet and four methods - Real-ESRGAN, SRGAN, U-Net and U-Net++ over 920nm, 930nm, 1000nm, 1030nm, 1064nm, 1100nm in 32-detector arrays data. The Fourier spectrogram is shown in the lower right.


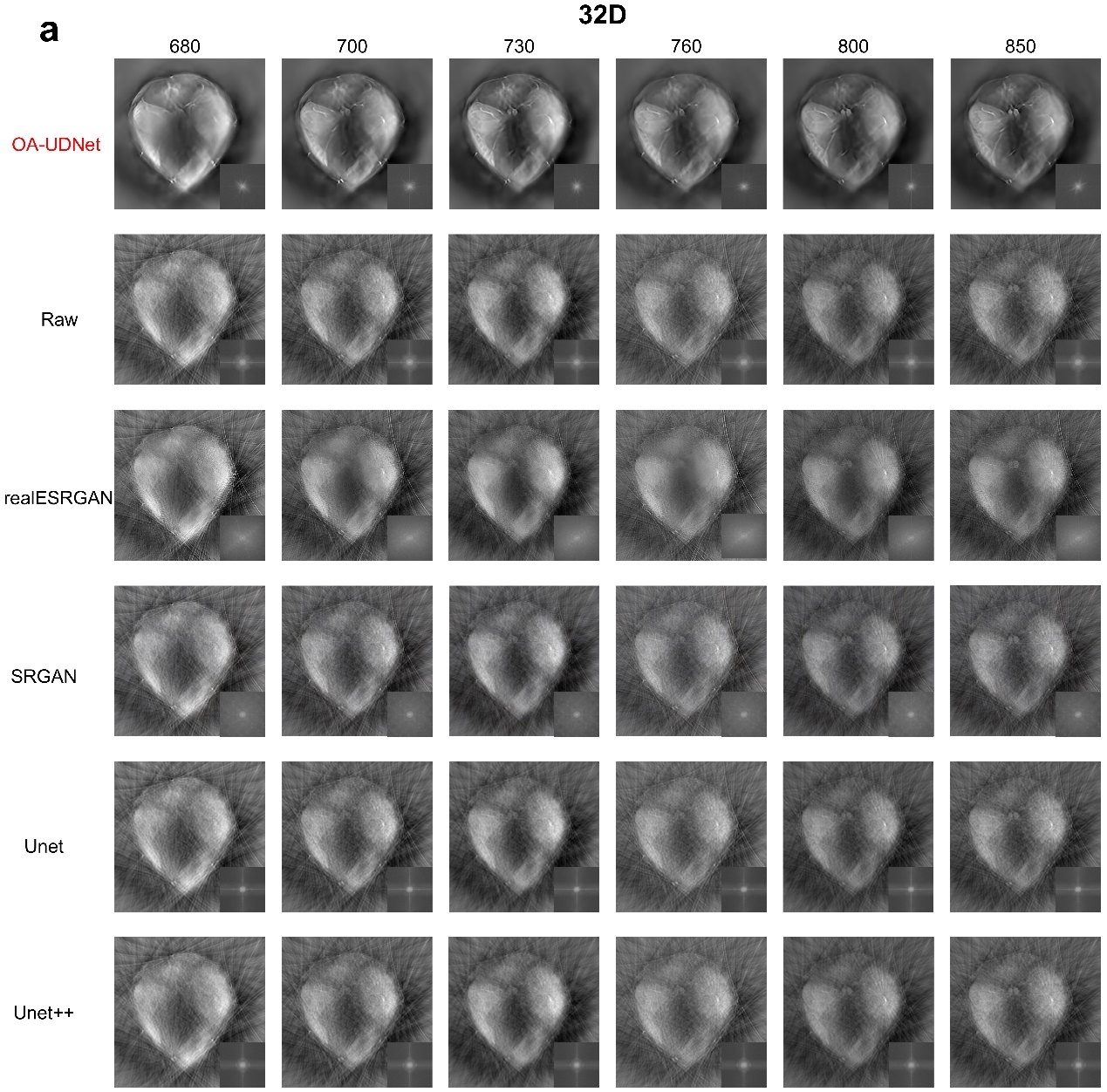


Supplementary Fig.S8: a Visual comparison of optoacoustic imaging (OAI) enhancement in the abdominal region achieved by OA-UDNet and four methods - Real-ESRGAN, SRGAN, U-Net and U-Net++ over 680nm, 700nm, 730nm, 760nm, 800nm, 850nm in 32-detector arrays data. The Fourier spectrogram is shown in the lower right.


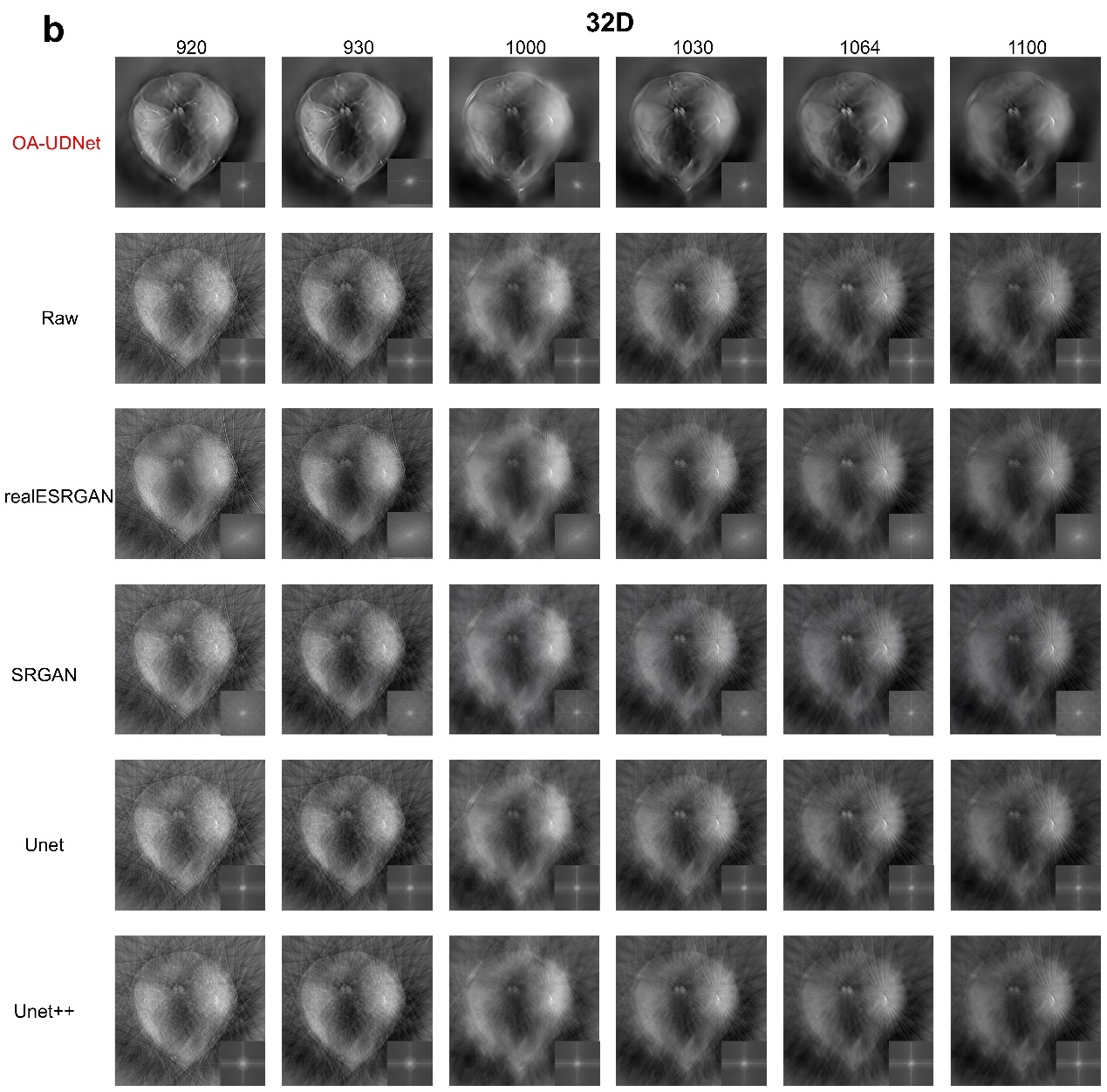
Supplementary Fig. S8: b Visual comparison of optoacoustic imaging (OAI) enhancement in the abdominal region achieved by OA-UDNet and four methods - Real-ESRGAN, SRGAN, U-Net and U-Net++ over 920nm, 930nm, 1000nm, 1030nm, 1064nm, 1100nm in 32-detector arrays data. The Fourier spectrogram is shown in the lower right.


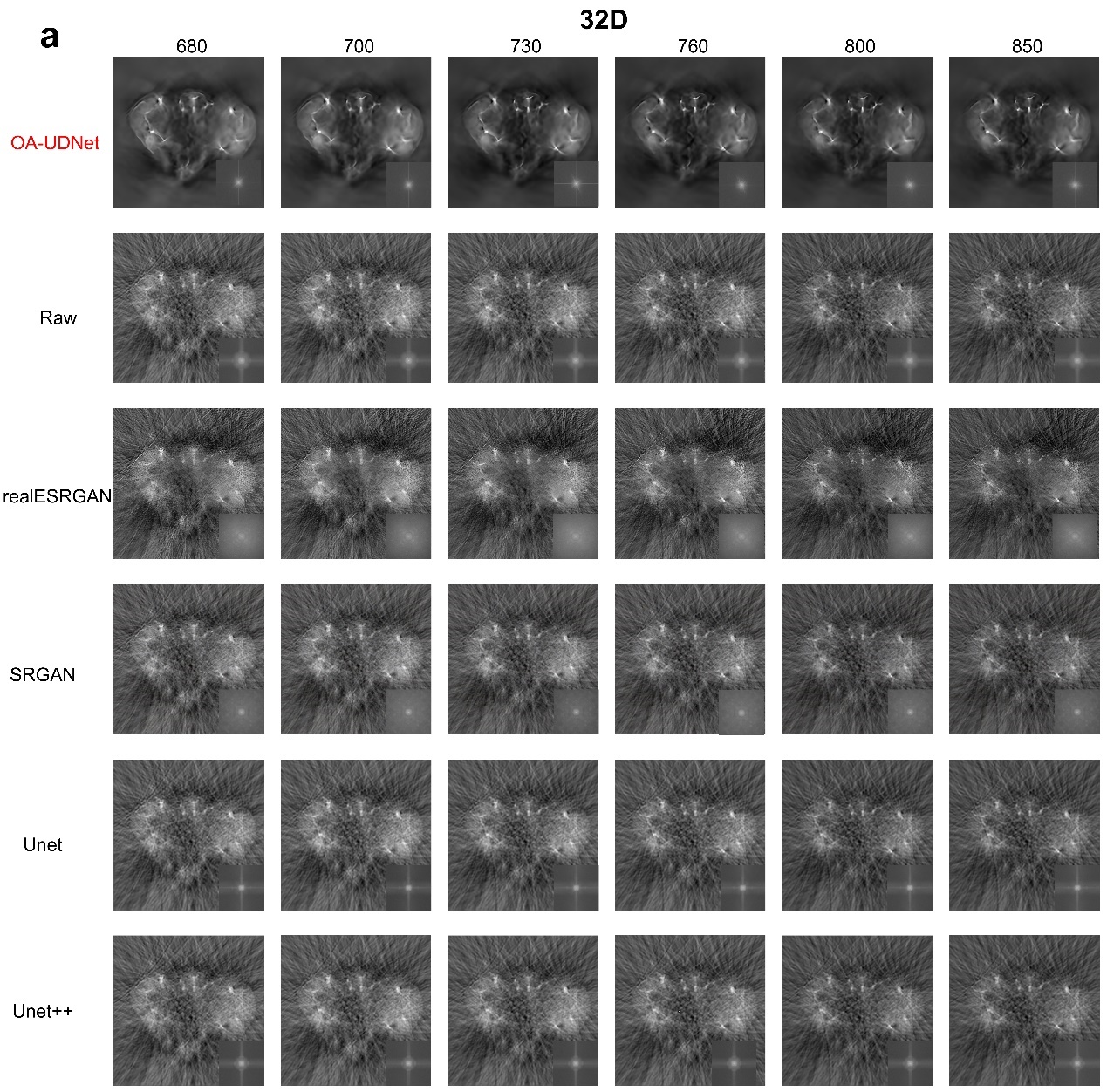


Supplementary Fig.S9: a Visual comparison of optoacoustic imaging (OAI) enhancement in the hindlimb region achieved by OA-UDNet and four methods - Real-ESRGAN, SRGAN, U-Net and U-Net++ over 680nm, 700nm, 730nm, 760nm, 800nm, 850nm in 32-detector arrays data. The Fourier spectrogram is shown in the lower right.


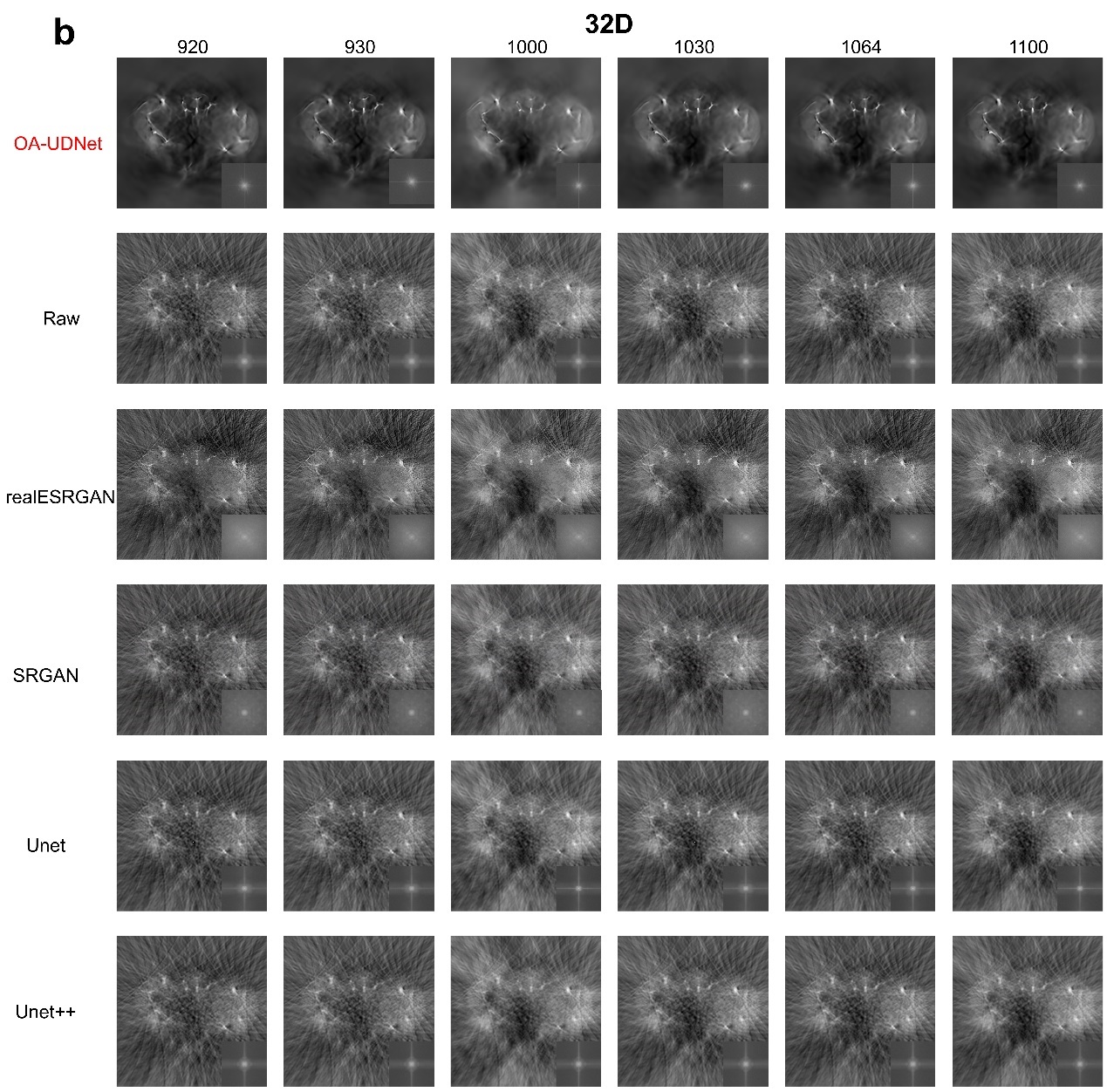


Supplementary Fig. S9: b Visual comparison of optoacoustic imaging (OAI) enhancement in the hindlimb region achieved by OA-UDNet and four methods - Real-ESRGAN, SRGAN, U-Net and U-Net++ over 920nm, 930nm, 1000nm, 1030nm, 1064nm, 1100nm in 32-detector arrays data. The Fourier spectrogram is shown in the lower right.


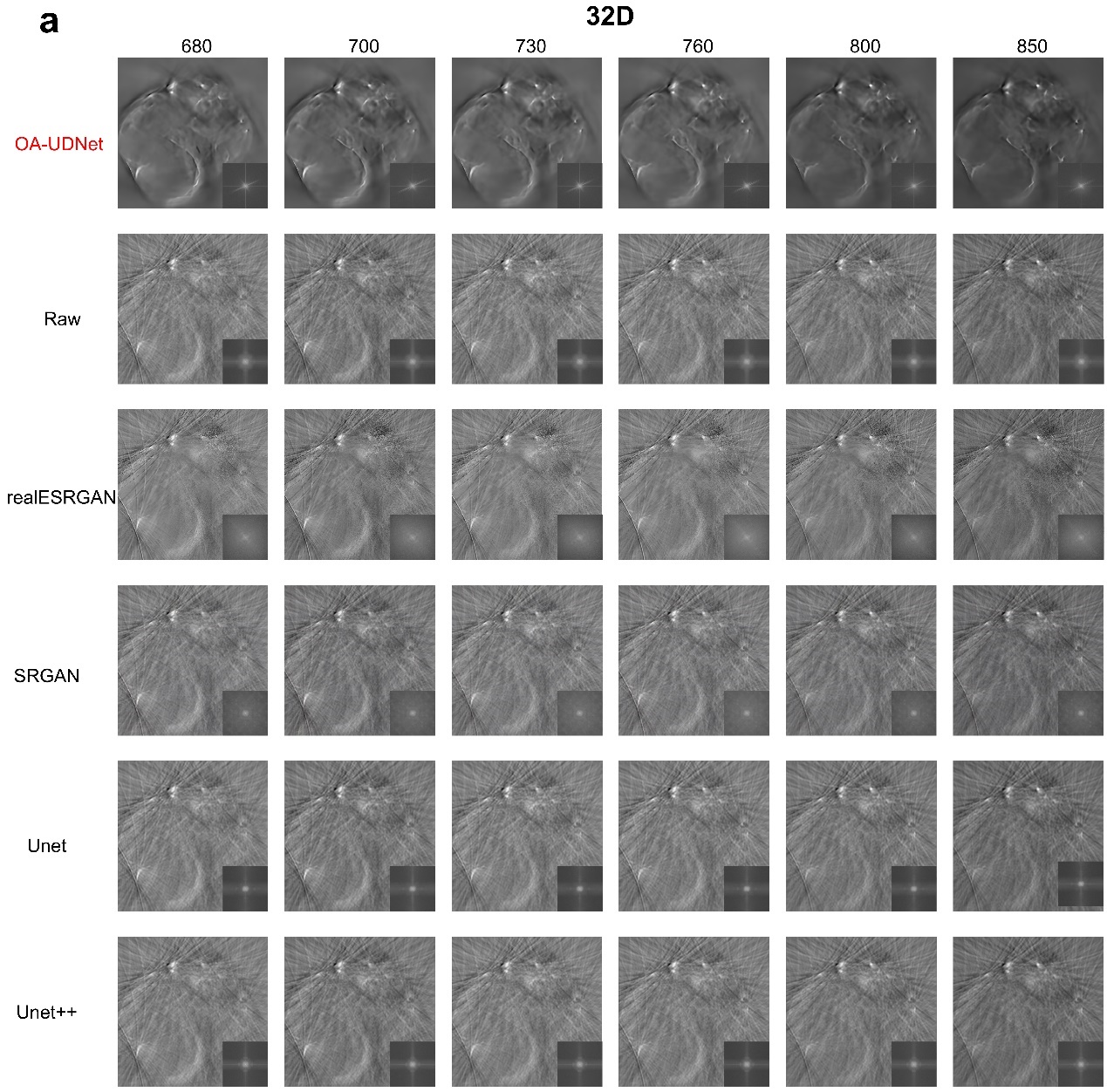


Supplementary Fig. S10: a Visual comparison of optoacoustic imaging (OAI) enhancement in the tumor region achieved by OA-UDNet and four methods - Real-ESRGAN, SRGAN, U-Net and U-Net++ over 680nm, 700nm, 730nm, 760nm, 800nm, 850nm in 32-detector arrays data. The Fourier spectrogram is shown in the lower right.


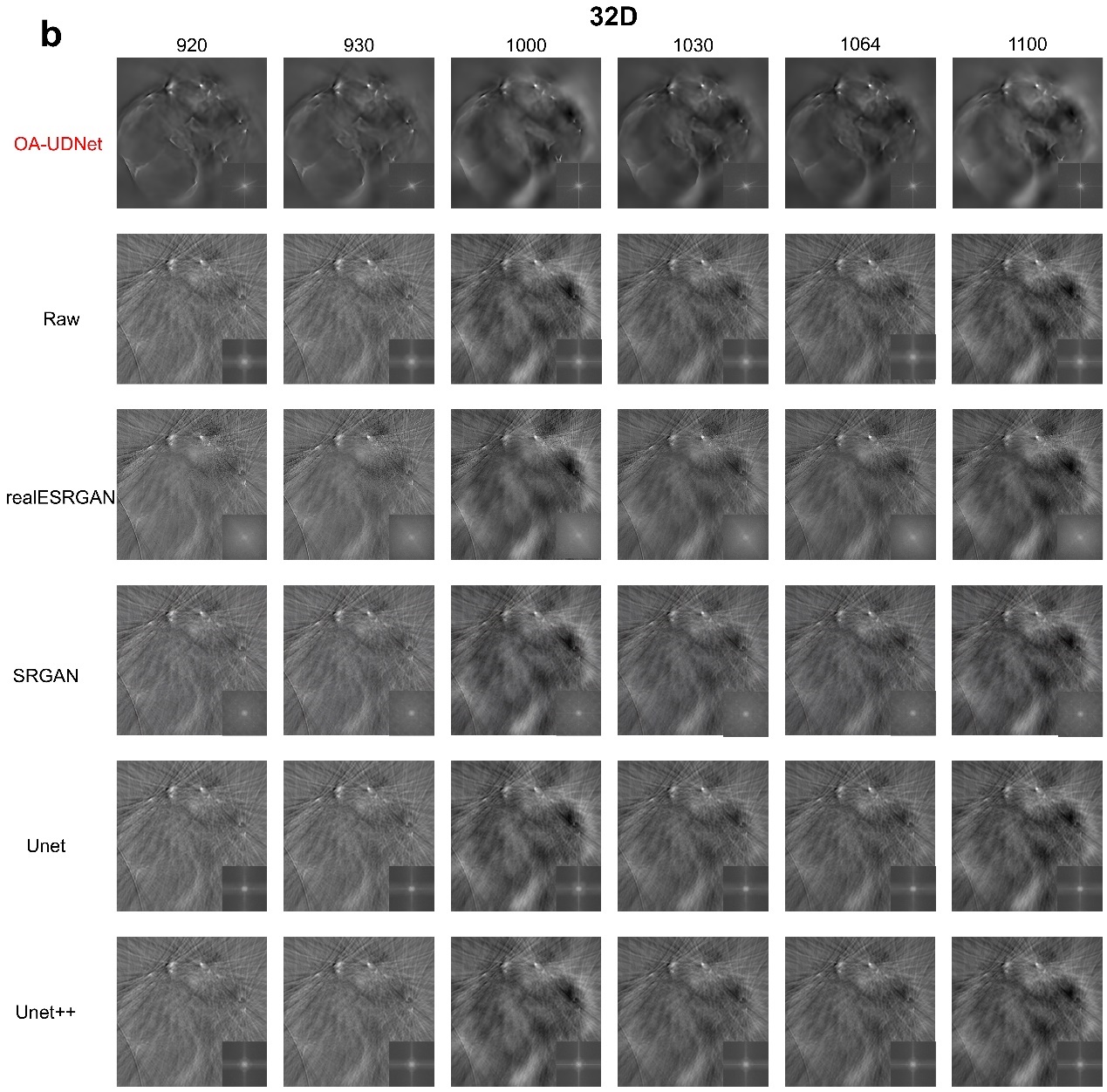


Supplementary Fig. S10: b Visual comparison of optoacoustic imaging (OAI) enhancement in the tumor region achieved by OA-UDNet and four methods - Real-ESRGAN, SRGAN, U-Net and U-Net++ over 920nm, 930nm, 1000nm, 1030nm, 1064nm, 1100nm in 32-detector arrays data. The Fourier spectrogram is shown in the lower right.

Supplementary Table S2. Quantitative statistical analysis of OA-UDNet, Real-ESRGAN, SRGAN, U-Net, and U-Net++ methods on the brain head dataset across 32-, 64-, and 128-detector arrays.

| Model comparison | Detector array | *P* value (PSNR) | *P* value (SSIM) | *P* value (LPIPS) |
| --- | --- | --- | --- | --- |
| OA-UDNet Vs. Raw | 32-Detectors | <0.001 | <0.001 | <0.001 |
| OA-UDNet Vs. Real-ESRGAN | 32-Detectors | <0.001 | <0.001 | <0.001 |
| OA-UDNet Vs. SRGAN | 32-Detectors | <0.001 | <0.001 | <0.001 |
| OA-UDNet Vs. U-Net | 32-Detectors | <0.001 | <0.001 | <0.001 |
| OA-UDNet Vs. U-Net++ | 32-Detectors | <0.001 | <0.001 | <0.001 |
| OA-UDNet Vs. Raw | 64-Detectors | <0.001 | <0.001 | <0.001 |
| OA-UDNet Vs. Real-ESRGAN | 64-Detectors | <0.001 | <0.001 | <0.001 |
| OA-UDNet Vs. SRGAN | 64-Detectors | <0.001 | <0.001 | <0.001 |
| OA-UDNet Vs. U-Net | 64-Detectors | <0.001 | <0.001 | <0.001 |
| OA-UDNet Vs. U-Net++ | 64-Detectors | <0.001 | <0.001 | <0.001 |
| OA-UDNet Vs. Raw | 128-Detectors | <0.001 | <0.001 | <0.001 |
| OA-UDNet Vs. Real-ESRGAN | 128-Detectors | <0.001 | <0.001 | <0.001 |
| OA-UDNet Vs. SRGAN | 128-Detectors | <0.001 | <0.001 | <0.001 |
| OA-UDNet Vs. U-Net | 128-Detectors | <0.001 | <0.001 | <0.001 |
| OA-UDNet Vs. U-Net++ | 128-Detectors | <0.001 | <0.001 | <0.001 |

Supplementary Table S3. Quantitative statistical analysis of OA-UDNet, Real-ESRGAN, SRGAN, U-Net, and U-Net++ methods on the mouse abdominal dataset across 32-, 64-, and 128-detector arrays.

| Model comparison | Detector array | *P* value (PSNR) | *P* value (SSIM) | *P* value (LPIPS) |
| --- | --- | --- | --- | --- |
| OA-UDNet Vs. Raw | 32-Detectors | <0.001 | <0.001 | <0.001 |
| OA-UDNet Vs. Real-ESRGAN | 32-Detectors | <0.001 | <0.001 | <0.001 |
| OA-UDNet Vs. SRGAN | 32-Detectors | <0.001 | <0.001 | <0.001 |
| OA-UDNet Vs. U-Net | 32-Detectors | <0.001 | <0.001 | <0.001 |
| OA-UDNet Vs. U-Net++ | 32-Detectors | <0.001 | <0.001 | <0.001 |
| OA-UDNet Vs. Raw | 64-Detectors | <0.001 | <0.001 | <0.001 |
| OA-UDNet Vs. Real-ESRGAN | 64-Detectors | <0.001 | <0.001 | <0.001 |
| OA-UDNet Vs. SRGAN | 64-Detectors | <0.001 | <0.001 | <0.001 |
| OA-UDNet Vs. U-Net | 64-Detectors | <0.001 | <0.001 | <0.001 |
| OA-UDNet Vs. U-Net++ | 64-Detectors | <0.001 | <0.001 | <0.001 |
| OA-UDNet Vs. Raw | 128-Detectors | <0.001 | <0.001 | <0.001 |
| OA-UDNet Vs. Real-ESRGAN | 128-Detectors | <0.001 | <0.001 | <0.001 |
| OA-UDNet Vs. SRGAN | 128-Detectors | <0.001 | <0.001 | <0.001 |
| OA-UDNet Vs. U-Net | 128-Detectors | <0.001 | <0.001 | <0.001 |
| OA-UDNet Vs. U-Net++ | 128-Detectors | <0.001 | <0.001 | <0.001 |

Supplementary Table S4. Quantitative statistical analysis of OA-UDNet, Real-ESRGAN, SRGAN, U-Net, and U-Net++ methods on the mouse hindlimb dataset across 32-, 64-, and 128-detector arrays.

| Model comparison | Detector array | *P* value (PSNR) | *P* value (SSIM) | *P* value (LPIPS) |
| --- | --- | --- | --- | --- |
| OA-UDNet Vs. Raw | 32-Detectors | <0.001 | <0.001 | <0.001 |
| OA-UDNet Vs. Real-ESRGAN | 32-Detectors | <0.001 | <0.001 | <0.001 |
| OA-UDNet Vs. SRGAN | 32-Detectors | <0.001 | <0.001 | <0.001 |
| OA-UDNet Vs. U-Net | 32-Detectors | <0.001 | <0.001 | <0.001 |
| OA-UDNet Vs. U-Net++ | 32-Detectors | <0.001 | <0.001 | <0.001 |
| OA-UDNet Vs. Raw | 64-Detectors | <0.001 | <0.001 | <0.001 |
| OA-UDNet Vs. Real-ESRGAN | 64-Detectors | <0.001 | <0.001 | <0.001 |
| OA-UDNet Vs. SRGAN | 64-Detectors | <0.001 | <0.001 | <0.001 |
| OA-UDNet Vs. U-Net | 64-Detectors | <0.001 | <0.001 | <0.001 |
| OA-UDNet Vs. U-Net++ | 64-Detectors | <0.001 | <0.001 | <0.001 |
| OA-UDNet Vs. Raw | 128-Detectors | <0.001 | <0.001 | <0.001 |
| OA-UDNet Vs. Real-ESRGAN | 128-Detectors | <0.001 | <0.001 | <0.001 |
| OA-UDNet Vs. SRGAN | 128-Detectors | <0.001 | <0.001 | <0.001 |
| OA-UDNet Vs. U-Net | 128-Detectors | <0.001 | <0.001 | <0.001 |
| OA-UDNet Vs. U-Net++ | 128-Detectors | <0.001 | <0.001 | <0.001 |

Supplementary Table S5. Quantitative statistical analysis of OA-UDNet, Real-ESRGAN, SRGAN, U-Net, and U-Net++ methods on the mouse tumor dataset across 32-, 64-, and 128-detector arrays.

| Model comparison | Detector array | *P* value (PSNR) | *P* value (SSIM) | *P* value (LPIPS) |
| --- | --- | --- | --- | --- |
| OA-UDNet Vs. Raw | 32-Detectors | <0.001 | <0.001 | <0.001 |
| OA-UDNet Vs. Real-ESRGAN | 32-Detectors | <0.001 | <0.001 | <0.001 |
| OA-UDNet Vs. SRGAN | 32-Detectors | <0.001 | <0.001 | <0.001 |
| OA-UDNet Vs. U-Net | 32-Detectors | <0.001 | <0.001 | <0.001 |
| OA-UDNet Vs. U-Net++ | 32-Detectors | <0.001 | <0.001 | <0.001 |
| OA-UDNet Vs. Raw | 64-Detectors | <0.001 | <0.001 | <0.001 |
| OA-UDNet Vs. Real-ESRGAN | 64-Detectors | <0.001 | <0.001 | <0.001 |
| OA-UDNet Vs. SRGAN | 64-Detectors | <0.001 | <0.001 | <0.001 |
| OA-UDNet Vs. U-Net | 64-Detectors | <0.001 | <0.001 | <0.001 |
| OA-UDNet Vs. U-Net++ | 64-Detectors | <0.001 | <0.001 | <0.001 |
| OA-UDNet Vs. Raw | 128-Detectors | <0.001 | <0.001 | <0.001 |
| OA-UDNet Vs. Real-ESRGAN | 128-Detectors | <0.001 | <0.001 | <0.001 |
| OA-UDNet Vs. SRGAN | 128-Detectors | <0.001 | <0.001 | <0.001 |
| OA-UDNet Vs. U-Net | 128-Detectors | <0.001 | <0.001 | <0.001 |
| OA-UDNet Vs. U-Net++ | 128-Detectors | <0.001 | <0.001 | <0.001 |


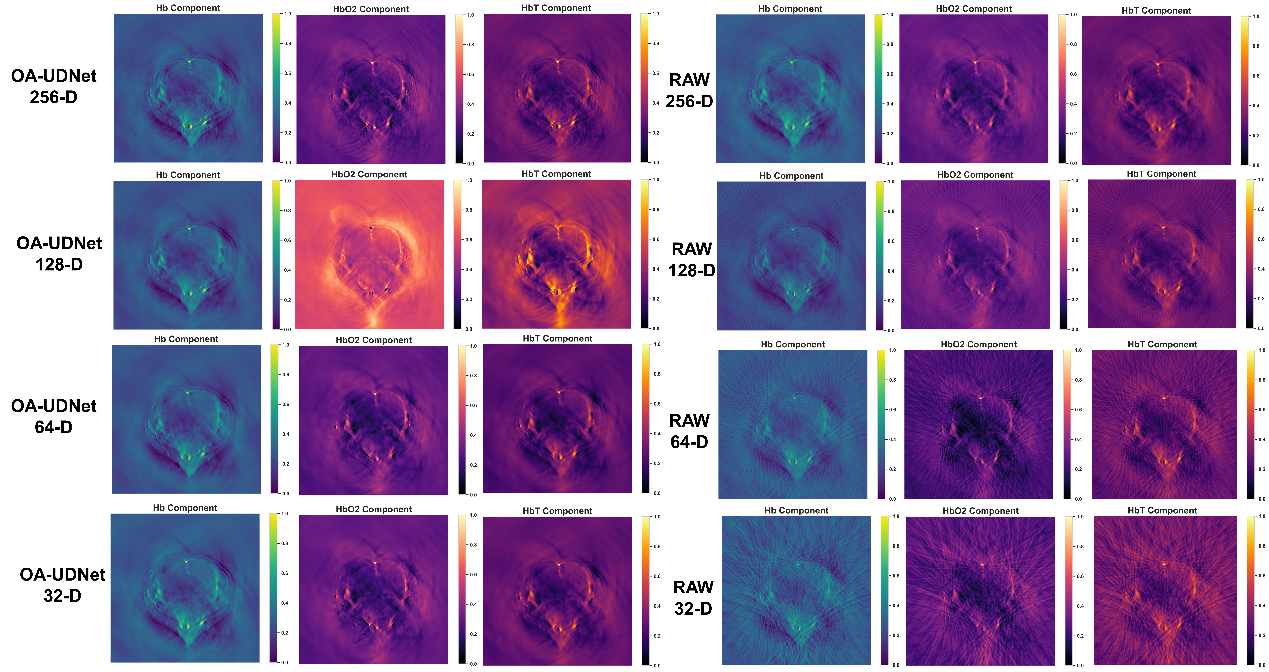


Supplementary Fig**. S11.** In vivo functional recovery of the mouse brain under varying sparse-sampling configurations using OA-UDNet.


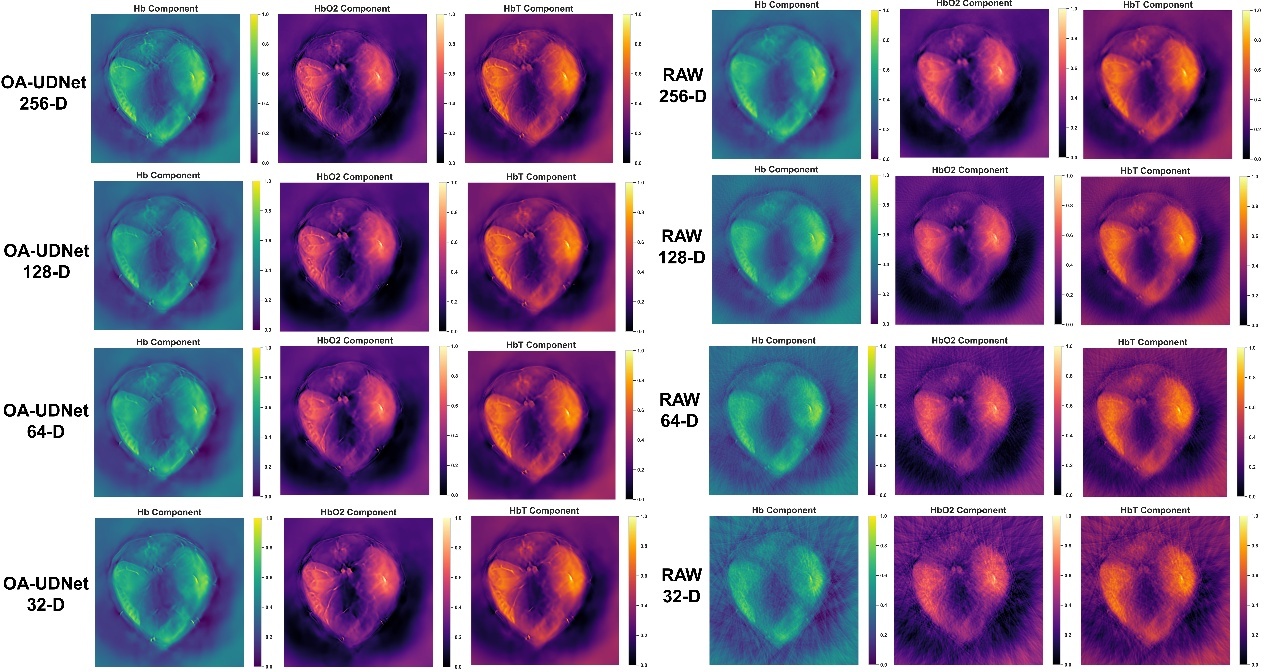


Supplementary Fig. **S12.** In vivo functional recovery of the mouse Abdomen under varying sparse-sampling configurations using OA-UDNet.


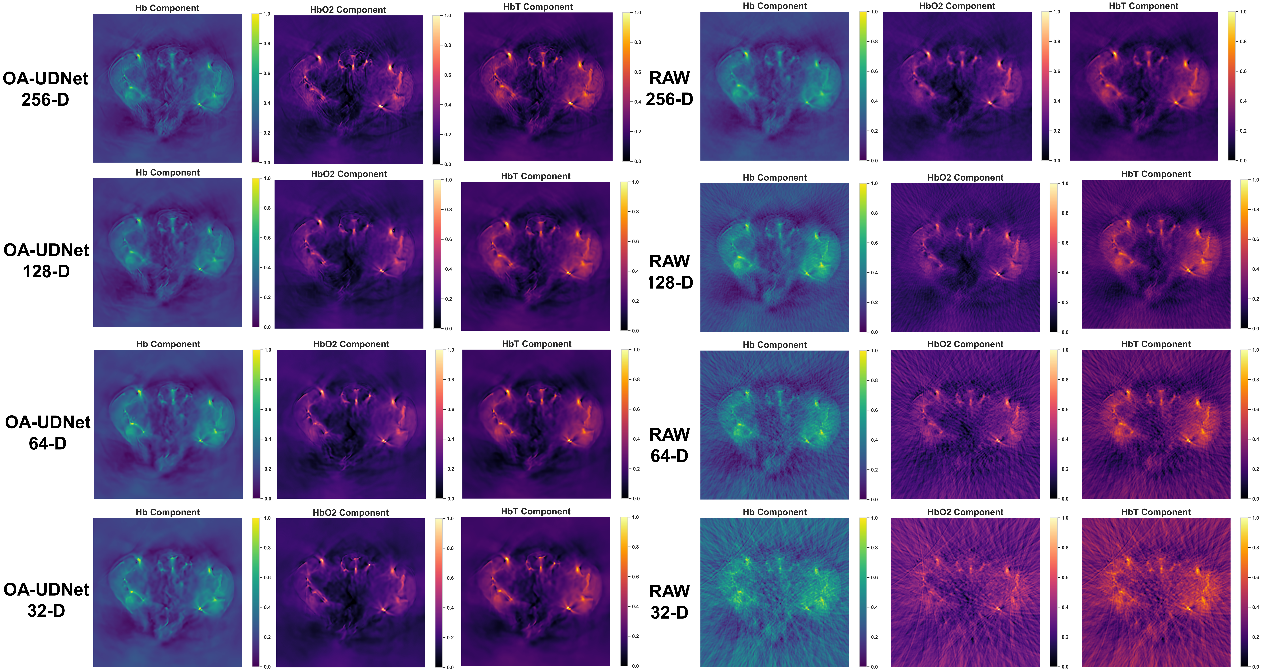


Supplementary Fig. **S13.** In vivo functional recovery of the mouse hindlimb under varying sparse-sampling configurations using OA-UDNet.


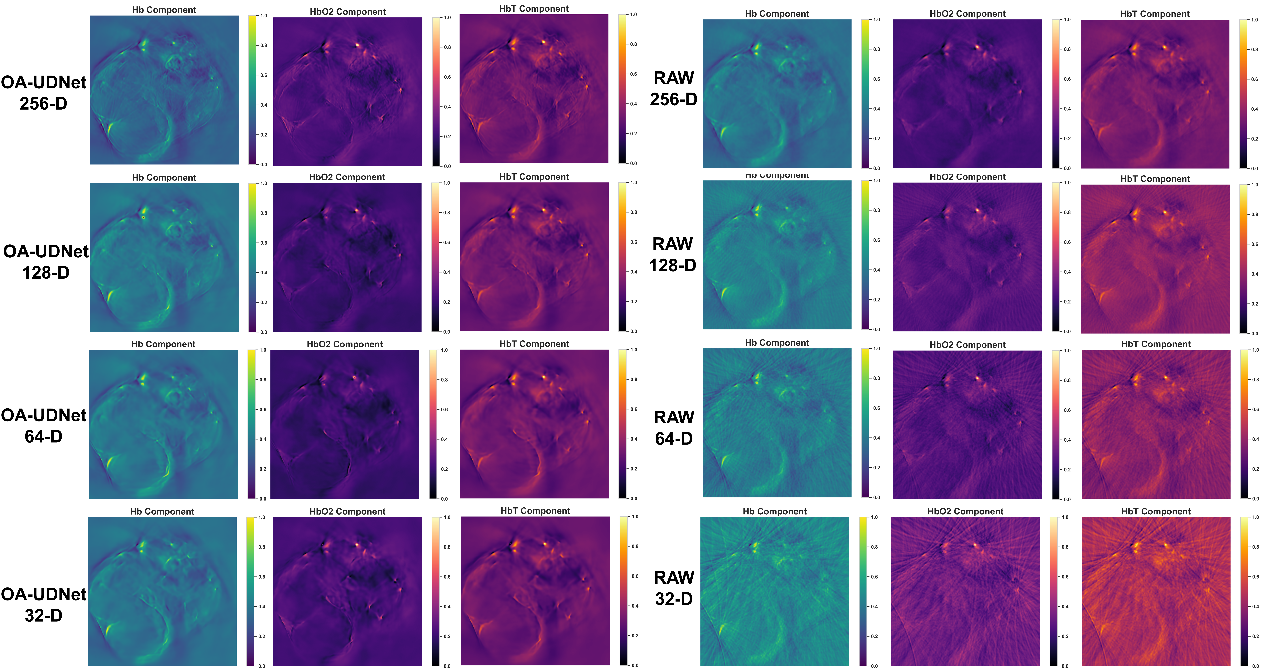


Supplementary Fig**. S14.** In vivo functional recovery of the mouse tumor under varying sparse-sampling configurations using OA-UDNet.

Supplementary **Table S6.** Global average Normalized Root Mean Square Error (NRMSE) of functional biomarker recovery across all anatomical regions under varying sparse-sampling conditions.

| Detector | Component | Raw Error (NRMSE, %) | OA-UDNet Error (NRMSE, %) | Absolute Error Reduction (%-points) |
| --- | --- | --- | --- | --- |
| 32 | HbO₂ | 7.97 | 1.56 | ↓ 6.42 |
| 32 | HbT | 8.85 | 1.58 | ↓ 7.27 |
| 32 | Hb | 6.7 | 1.48 | ↓ 5.22 |
| 64 | HbO₂ | 4.76 | 1.85 | ↓ 2.91 |
| 64 | HbT | 4.53 | 1.73 | ↓ 2.80 |
| 64 | Hb | 3.74 | 1.72 | ↓ 2.01 |
| 128 | HbO₂ | 2.77 | 1.68 | ↓ 1.09 |
| 128 | HbT | 3.27 | 1.68 | ↓ 1.60 |
| 128 | Hb | 3.69 | 2.4 | ↓ 1.30 |

Supplementary **Table S7**. Quantitative performance comparison on the human dataset under varying sparse-sampling configurations.

|  | Detector | PSNR (dB) | SSIM |
| --- | --- | --- | --- |
| OA | 32 | 28.4425 ± 0.2555 | 0.8412 ± 0.0300 |
| OA | 64 | 30.3755 ± 0.3067 | 0.8425 ± 0.0240 |
| OA | 128 | 33.0588 ± 0.5651 | 0.8344 ± 0.0217 |
| Raw | 32 | 21.9860 ± 0.4388 | 0.6649 ± 0.0033 |
| Raw | 64 | 25.8680 ± 0.3890 | 0.6940 ± 0.0029 |
| Raw | 128 | 31.3563 ± 0.2890 | 0.7352 ± 0.0022 |


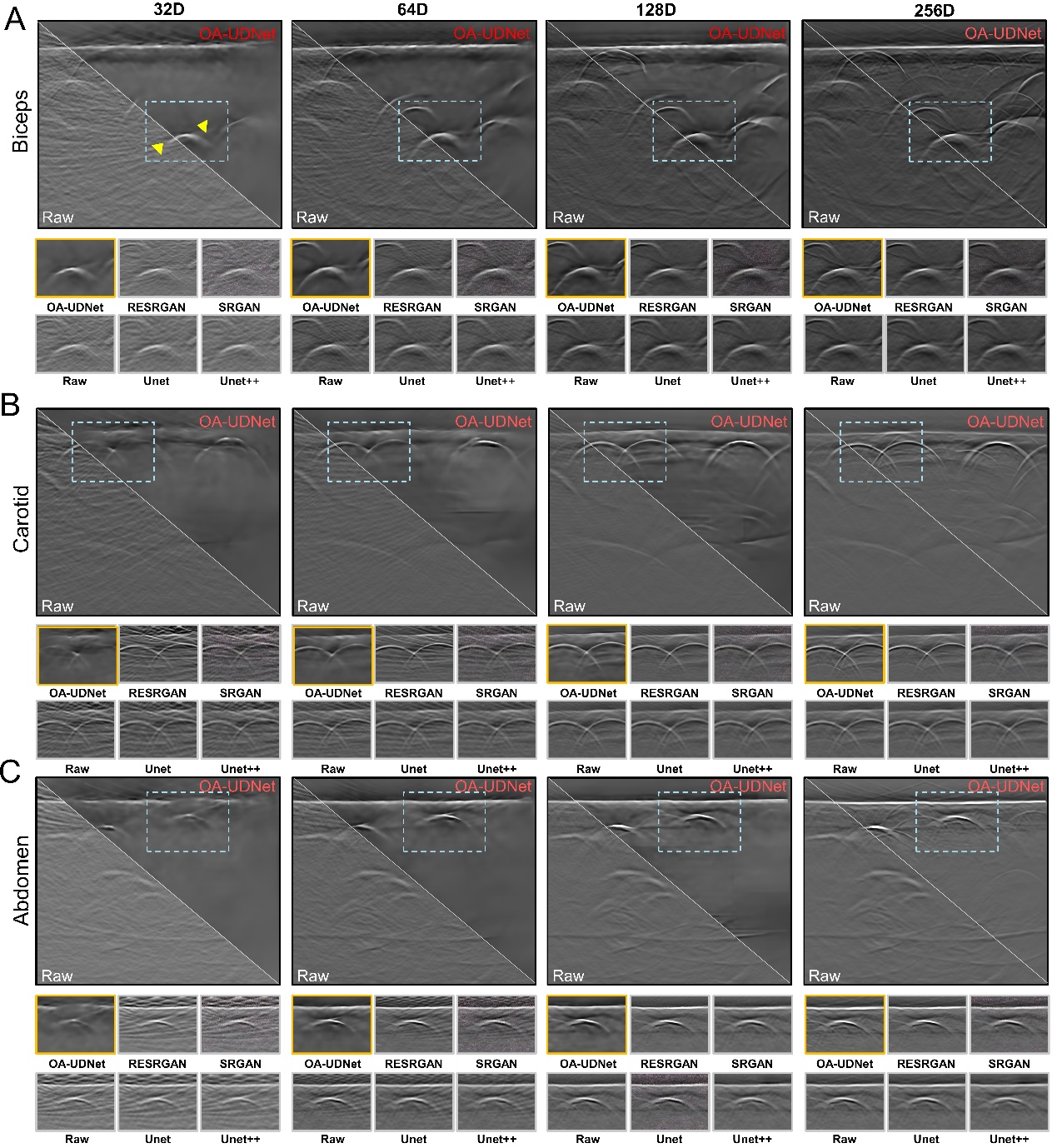


**Fig. S15.** Validation of OA-UDNet on human optoacoustic imaging (OAI).

(a–d) Visual comparison of OAI enhancement achieved by OA-UDNet and four benchmark methods—Real-ESRGAN, SRGAN, U-Net, and U-Net++—at 800 nm, for the human dataset. Comparisons are presented between OA-UDNet-enhanced OAI, raw OAI, and the enhanced versions using Real-ESRGAN, SRGAN, U-Net, and U-Net++. Blue boxes highlight notable improvements in image detail and contrast.

Supplementary **Table S8.** Quantitative performance comparison between the standard DDPM and the proposed OA-UDNet under the extremely sparse 32-detector configuration.

| Datasets | Metrics | Standard DDPM | OA-UDNet |
| --- | --- | --- | --- |
| Abdomen | PSNR (dB) | 17.76 ± 3.60 | 36.56 ± 2.11 |
|  | SSIM | 0.861 ± 0.050 | 0.979 ± 0.005 |
| Brain | PSNR (dB) | 19.23 ± 2.99 | 38.99 ± 1.91 |
|  | SSIM | 0.868 ± 0.036 | 0.983 ± 0.004 |
| Hindlimb | PSNR (dB) | 21.08 ± 2.82 | 31.95 ± 4.88 |
|  | SSIM | 0.887 ± 0.043 | 0.965 ± 0.018 |


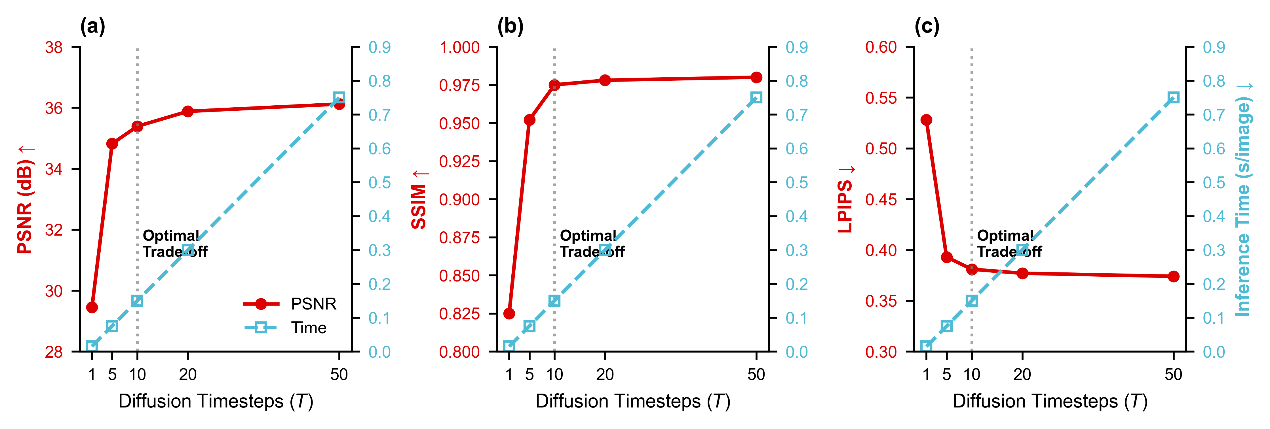


**Fig. S16.** Optimization of diffusion timesteps in OA-UDNet. Quantitative evaluation of reconstruction quality and computational cost across varying diffusion steps (T in {1, 5, 10, 20, 50}). a, Peak signal-to-noise ratio (PSNR). b, Structural similarity index measure (SSIM). c, Learned perceptual image patch similarity (LPIPS). The red solid lines denote the imaging metrics (left axis), while the blue dashed lines represent the linear scaling of inference time per image on an RTX 4090 GPU (right axis). Increasing the steps from 1 to 10 significantly improves all quality indicators (e.g., PSNR increases from 29.45 dB to 35.390 dB). Extending the diffusion chain to 20 or 50 steps yields negligible marginal gains but substantially increases latency. The vertical dotted line marks T = 10, which provides the optimal algorithmic balance between high-fidelity structural recovery and high-throughput computational speed (0.15 s/image).

**Methods section**

Supplementary Note 4

Experimental protocols, and signal pre-processing.

The MSOT system emits multi-wavelength pulses (660–1300 nm) at 20 Hz and uses a 256-element transducer array with 5 MHz center frequency. Animal holders and coupling with purified water ensure proper alignment and acoustic transmission.

Tumor-bearing mice were anesthetized with 1% pentobarbital sodium (0.01 mL/g), and tumors were kept below 1.5 cm in diameter per ethical requirements (approval number: 2024-FAET-001, Fudan University). Whole-body scans cover the head, abdomen, hindlimbs, and tumor sites. Multispectral acquisition included wavelengths: 680, 700, 730, 760, 800, 850, 920, 1000, 1030, 1064, 1100 nm.

For image reconstruction, signals were band-pass filtered (0.5–8 MHz for animals; 1.5–10 MHz for human data) prior to back-projection. Sparse sampling data were obtained by uniform down-sampling at 1/2, 1/4, and 1/8 ratios to simulate 128, 64, and 32 detectors, enabling evaluation of network performance under reduced detector configurations.

Supplementary Note 5

Local Contrast Calculation:

For each pixel at position $(x,y)$, define a surrounding window of size k x k. Within this window, compute the local contrast $C(x,y)$ using the formula:

$$C(x,y)=\frac{I_{max}-I_{min}}{\mu+\epsilon}$$

Where:

$I_{max}$ is the maximum intensity value within the window.

$I_{min}$is the minimum intensity value within the window.

$\mu$ is the mean intensity value within the window.

$\epsilon$ a small constant to prevent division by zero.

Average Local Contrast Calculation: After computing $C(x,y)$ for all pixels, the Average Local Contrast $\overline{C}$ is determined by averaging these values over the entire image.

$$\overline{C}=\frac{1}{M\times N}\sum_{x=1}^{M} \sum_{y=1}^{N} C(x,y)$$

Where: M and N are the dimensions of the image.

Supplementary Note 6 Average Energy of Gradient:

The Average Energy of Gradient (EOG) is a metric used to assess the sharpness or clarity of an image by evaluating the intensity variations between adjacent pixels. A higher EOG value typically indicates a sharper image. The EOG is calculated using the following formula:

$$EOG=\frac{1}{M\times N}\sum_{x=1}^{M} \sum_{y=1}^{N} [(I(x+1,y)-I(x,y))^{2}+(I(x,y+1)-I(x,y))^{2}]$$

Where: $I(x,y)$represents the pixel intensity at position $(x,y)$.

M and N denote the width and height of the image, respectively
